# Influence of Genetic Polymorphism Towards Pulmonary Tuberculosis Susceptibility

**DOI:** 10.3389/fmed.2018.00213

**Published:** 2018-08-16

**Authors:** Murugesan Harishankar, Paramasivam Selvaraj, Ramalingam Bethunaickan

**Affiliations:** Department of Immunology, National Institute of Research in Tuberculosis, Chennai, India

**Keywords:** *Mycobacterium tuberculosis*, genetic polymprhisms in TB, HLA genes, Non HLA genes, host genetics, cytokine and chemokine polymorphisms

## Abstract

Tuberculosis (TB) is still remains the major threat for human health worldwide. Several case-control, candidate-gene, family studies and genome-wide association studies (GWAS) suggested the association of host genetic factors to TB susceptibility or resistance in various ethnic populations. Moreover, these factors modulate the host immune responses to tuberculosis. Studies have reported genetic markers to predict TB development in human leukocyte antigen (HLA) and non-HLA genes like killer immunoglobulin-like receptor (KIR), toll-like receptors (TLRs), cytokine/chemokines and their receptors, vitamin D receptor (VDR) and SLC11A1 etc. Highly polymorphic HLA loci may influence antigen presentation specificities by modifying peptide binding motifs. The recent meta-analysis studies revealed the association of several HLA alleles in particular class II HLA-DRB1 with TB susceptibility and valuable marker for disease development especially in Asian populations. Case-control studies have found the association of HLA-DR2 in some populations, but not in other populations, this could be due to an ethnic specific association of gene variants. Recently, GWAS conducted in case-control and family based studies in Russia, Chinese Han, Morocco, Uganda and Tanzania revealed the association of genes such as *ASAP1, Alkylglycerol monooxygenase (AGMO), Forkhead BoxP1 (FOXP1), C-terminal domain phosphatase 1* (*UBLCP1*) and intergenic SNP rs932347C/T with TB. Whereas, SNP rs10956514A/G were not associated with TB in western Chinese Han and Tibetan population. In this review, we summarize the recent findings of genetic variants with susceptibility/resistance to TB.

## Introduction

Tuberculosis (TB) remains a serious global health problem with approximately 5–10% latent tuberculosis infections (LTBI) individuals and among which 90–95% individuals will develop active tuberculosis infection. According to WHO Report 10.4 million new TB cases and 1.3 million deaths occurred in 2016 (1). Host genetic factors play an important role in determining inter-individual difference in susceptibility or resistance to infectious diseases including TB. However, the mechanisms involved in restriction of disease development in latent infection or lead to severe active disease are still largely unknown. Studies such as case-control, twin, candidate gene approaches, family-based and genome-wide association studies (GWAS) revealed the association of genetic factors with susceptibility or resistance to TB (2–8). Twin studies revealed higher TB susceptibility rate in monozygotic twins than dizygotic twins or siblings (9, 10). Immune response to *Mycobacterium tuberculosis* (Mtb) involves several important genes such as human leukocyte antigen (HLA) and non-HLA genes like killer immunoglobulin-like receptor (KIR), toll-like receptors (TLRs), cytokine/chemokines and their receptors, vitamin D receptor (VDR), SLC11A1 and C-type lectins etc. Polymorphisms in these genes may regulate the expression and have a diverse influence on the susceptibility or protection against TB among particular families, ethnicities and races. The identification of host genetic markers will be useful to predict the development or predisposition to develop TB and understanding the immunopathogenesis of the disease. Moreover, protective HLA alleles to TB will be useful in new epitope based vaccine development. Several methodologies have been employed for genotyping different SNPs. The polymerase chain reaction (PCR) based methods has made possible to study HLA and non-HLA gene polymorphisms at DNA level. This molecular technology has gradually replaced the HLA serotyping and cellular typing methods which had several limitations compared to DNA based methods. The common methods are PCR based sequence specific primers (PCR-SSP), PCR based restriction fragment length polymorphism (PCR-RFLP), single-strand conformation polymorphism (PCR-SSCP), sequence-specific oligonucleotide (PCR-SSO) and single nucleotide polymorphism (PCR-SNP). Recently, exome sequencing, pyro-sequencing and next generation sequencing (NGS) technology has emerged as a simpler and more informative than classical molecular methods and employs DNA amplification followed by sequencing. In this review, we summarize the association of HLA and non-HLA gene polymorphisms in different ethnic populations and current advanced molecular methods for SNP genotyping.

## Genetic variants and TB association

### Mendelian susceptibility to mycobacterial diseases (MSMD)

Mendelian susceptibility to mycobacterial diseases is a clinical disorder in whom familial susceptibility to mycobacterial disease is observed. It is characterized by a narrow vulnerability to poorly virulent mycobacteria, such as bacillus Calmette-Guérin (BCG) vaccines and environmental mycobacteria. Only about half of patients with MSMD have an identified genetic cause. The pattern of heritability differs according to the gene affected and the specific genetic lesion. Nine genes are known to be responsible for MSMD. Seven of them are inherited in an autosomal recessive or autosomal dominant pattern IFNGR1 (OMIM 209950, 615978, 600263, 610424, 607948; 6q23.3), IFNGR2 (OMIM 614889; Chr21q22.11), STAT1 (OMIM 614892, 613796, 614162; Chr2q32.2), IL12RB1 (OMIM 614891, Chr19q13.11), IL12B (5q33.3), IRF8 (OMIM 614893-4; Chr16q24.1), ISG15 (OMIM 61626; Chr1p36.33), TYK2 (OMIM 611521, Chr19p13.2) and 2 less severe X-linked (IKBG/NEMO (OMIM 300636, ChrXq28) and CYBB (also known as p91-phox, OMIM 306400, 300545; ChrXp21.1-p11.4) recessive mutations (11–19). The genes implicated in MSMD have frequently been studied through linkage and candidate gene studies which cumulatively indicate that genes involved in MSMD do not appear to account for non-familial cases of TB (20).

### Candidate gene studies

Candidate gene studies includes SNPs in a specific gene, which implicated to association with particular disease.

### Human leukocyte antigen (HLA)

Human leukocyte antigen is the most polymorphic loci located in chromosome 6 of the human genome. Polymorphisms in Class I and Class II genes may alter the binding affinity of antigens which could lead to impaired antigenic presentation to CD4+ and CD8+ T-cells. Among the class I and class II alleles HLADRB1 is the most associated with TB susceptibility/protection. A meta-analysis conducted in China reported that DRB1^*^03 and DRB1^*^07 may provide protective effects against TB susceptibility (21). Another meta-analysis of 31 studies revealed the association of HLADRB1 ^*^04, ^*^09, ^*^10, ^*^15, and ^*^16 with TB risk, particularly in East Asian population while HLADRB1^*^ 11 associated with protective to TB (22). In Uganda study, HLA-DQB1^*^03:03 allele was associated with protection to TB (23). In contrary, Amazon Brazilian population DRB1^*^04:07:01, DRB1^*^04:11:01, and DRB1^*^04:92 of the HLA-DRB1^*^04 associated with susceptibility to TB (24). A recent study conducted among 682 TB patients and 836 healthy controls in Thailand reported that HLA-DRB1^*^09:01 and HLA-DQB1^*^03:03 alleles were associated with TB susceptibility in patients infected with modern Mtb strains and suggested strain specific susceptibility to TB (25). In general, meta-analysis and other recent studies suggested the inconsistent results among different ethnic groups this could be due to population heterogeneity and small sample size. Hence well-designed studies with larger sample sizes are needed to confirm HLA association in TB. Various HLA studies in different ethnic populations given in Table 1.

**Table 1:** Association between HLA and TB in different ethnic population.

**Ethnicity/Country**	**Allele/Haplotype**	**Association**	**Sample size**		**References**
			**HCs**	**TB**	
Asian/India	HLA-DR2	Susceptibility	287	143	(26)
	HLAB^*^57	Protective in female HIV patients	682	238	(27)
	HLA-DRB1^*^1501 and DQB1^*^0601	Susceptibility	87	126	(28)
	DPB1^*^04	Protective			
	DRB1^*^1501	Susceptibility	46	20	(29)
	DRB1^*^1501-DRB5^*^0101-DQA1^*^0103-DQB1^*^0601(Haplotype)	Susceptibility			
	HLA-DR2	Susceptibility	289	153	(30)
	DRB1^*^1501 and DRB1^*^1502				
	A2	Susceptibility	329	153	(31)
	B18	Protective			
	HLA-DRB1^*^14, DQB1^*^0503 and DQB1^*^0502	Susceptibility	–	–	(32)
	DRw6	Protective	109	124	(33)
	DR2	Susceptibility	–	25 families	(34)
	A1- like supertype	Protective	289	235	(35)
	A3- like supertype	Susceptibility			
	HLA-DRB1^*^15	Susceptibility	178	210	(36)
	A^*^24-B^*^40-DRB1^*^15				
	HLA-DRB1^*^16	Protection			
	A^*^02-B^*^40-DRB1^*^16 and A^*^02-B40-DRB1^*^03				
Asian/India	HLA-DR2 DQ1	Susceptibility	122	209	(37)
	DRB1^*^1501(DR2)	Susceptibility (inverse correlation to lymphocyte proliferation response)	36	72	(38)
		Downregulation of perforin positive CTL and NK cells			(39)
	Haplotypes	Susceptibility to TB in HIV			(40)
	HLA-DRB1^*^1502-DPB1^*^0201				
	HLA-DQB1^*^0601-DPB1^*^0201				
	HLA-DRB1^*^1502-DQB1^*^0601-DPB1^*^0201				
	HLA-A11,1101	Protective to HIV and HIV-TB			(41, 42)
	HLA-B40, 4006	Susceptibility to HIV and HIV-TB			
	HLA-DR2	Susceptibility to HIV and HIV-TB			
	HLA-DRB1^*^1502	Susceptibility to HIV-TB			(41)
	HLA-DQB1^*^050301	Susceptibility to HIV and HIV-TB			
	HLA-DPB1^*^1501	Protective to TB and HIV-TB			
	HLA-DRB1^*^13, HLA-DRB5 and HLA-DQB1^*^06	Susceptibility to TB in HIV	50	50	(43)
	HLA DQB1^*^02	Protective to TB in HIV			
	HLA-B^*^15	Susceptibity to TB in HIV			
	HLA-B^*^51	Protective to TB in HIV			
Asian/China	HLA-DR,DQ	Susceptibility to bone TB	88	86	(44)
	HLA-DRB1 ^*^ 15 HLA-DRB1 ^*^ 11	SusceptibilityProtective	90	74	(45)
	DRB1 ^*^ 09	Susceptibility to PTB with type2 diabetes mellitus	46	123	(46)
	DQB1 ^*^ 05	Protective to PTB with type2 diabetes mellitus			
	DR1 and DR13.3	Protective to PTB	101	110	(47)
	HLA-DR and DQ	Susceptibility	146	146	(48)
	HLA-DRB	Susceptibility	62	97	(49) (50)
	HLA-DRB1	Susceptibility	105	80	
	HLA-DRB1	Susceptibility	231	226	(51)
	HLA-DRB1, DQA1 and DQB1	Susceptibility	189	176	(52)
	HLA-DRB1^*^04 and HLA-DQB1^*^0201	Susceptibility	230	231	(53)
Asian/Korea	DRB1^*^0803 and DQB1^*^0601	Susceptibility	200	160	(54)
Asian/Combodia	HLA-DQB1^*^0503	Susceptibility	88	126	(55)
African/ SouthAfrica	DRB1^*^1302	Susceptibility	117	95	(56)
	DRB1^*^1302- DQB1^*^0602/3(Haplotype) DQB1^*^0301-0304(Phenotype) DQB1^*^0301-0304/DRB1^*^1101-1121(Haplotype) DRB1^*^1101-1121-DQB1^*^05(Haplotype)				
African/Uganda	HLA-DQB1^*^03:03		42	43	(23)
Caucasian/ Mexico	DQA1^*^0101,DQB1^*^0501 and DRB1^*^1501	Susceptibility	95	50	(57)
	DQB1^*^0402, DR4 and DR8	Protection			
Caucasian/Iran	HLA-DRB1^*^07 and HLA-DQA1^*^0101	Susceptibility	100	40	(58)
	HLA-DQA1^*^0301 and 0501	Protective			
	A26 and B27 B17 and DR14	Protective Susceptibility	108	44	(59)
Caucasian/Syria	DRB1^*^04 DRB1^*^11	Susceptibility Protective	209	147	(60)
Caucasian/ Portugal	HLA-DRB1^*^14	Susceptibility	82	92	(61)
Caucasian/Greek	HLA-A R^114^and HLA-DRβN^37^(Aminoacid poly)	Susceptibility	46	86	(62)
	HLA-A S^77^	Protection			
Caucasian/Brazil	HLA-A^*^02 and HLA-B^*^18	Protection	224	112	(63) (24)
Canadian/Canada	B8 HLA-A^*^03 and HLA-DQB1^*^05:03	Susceptibility Susceptibility	543 –	46 –	(64, 65)
Black American/United states	B5 and DR5 DR6	Susceptibility Protection	54	72	(66)
Italian/Italy	DR4 or along with B14 A2+,B14-,DR4-	Susceptibility Protection	1089	122	(67)
Indonesian/ Indonesia	DR2 and DQw1 DQw3	Susceptibility Protection	64	101	(68)
Polish/Poland	DRB1^*^13 DRB1^*^16 DRB1^*^05DRB1^*^1601-DQB1^*^0502, DRB1^*^04-DQB1^*^03 and DRB1^*^14-DQB1^*^05 DRB1^*^11-DQB1^*^03	ProtectionSusceptibilitySusceptibilitySusceptibility	58 58125	313861	(69) (70) (71)
Thai/Thailand	HLA-DQB1^*^0502HLA-DQA1^*^0601 and DQB1^*^0301 HLA-DRB1^*^09:01 and HLA-DQB1^*^03:03	SusceptibilityProtectionSusceptibility	160 836	82 682	(72) (25)
Soviet Union (six Ethnic groups) / Russia	DR2 DR3	Susceptibility Protection	984	643	(73)
Iraqi Arab	HLA-B18 and HLA-DR1HLA-B5 and HLA-DR8	Susceptibility Protection	40	105	(74)

### Toll like receptors (TLRs)

TLRs are pattern recognition receptors play an important role in host innate immune responses and activate adaptive immunity by inducing production of inflammatory cytokines and antimicrobial molecules by T helper (Th1) cells in response to Mtb infection (75–77). Different TLRs such as TLR-2, 4, 8, and 9 play an important role in Mtb infection (78). Among the 10 TLRs, TLR2 and TLR4 interact with mycobacterial lipoprotein and whole live Mtb to mediate immune responses (79, 80). Association with TB was reported in several studies and found diverse among various ethnic groups (81, 82). In a case-control study conducted in a South African population investigated 23 polymorphisms in TLR genes. The results suggested the association of TLR8 polymorphism (rs3761624A/G) with TB susceptibility in females compared to males. Whereas, inTLR1 (rs5743618G/T) and TLR8 (rs3764879C/G and rs3764880A/G) both sexes were associated with susceptibility to TB (83). A study conducted in Pakistani population, TLR8 “G” allele (rs3764880A/G) associated with susceptibility to TB and with bacterial load. Moreover, this study revealed a strong association among males (84). In contrary, cohort study from Hyderabad, India reported that “A” allele of TLR1-248 Ser/Asn (rs4833095 G/A) associated with TB protection and evoke a better immune response by activation of TNF-α production and transcription factor NF-kB in Mtb lysate infected peripheral blood mononuclear cells (PBMC) and human embryonic kidney (HEK) cells *in vitro* (85). Another case-control study from Lucknow, India reported that TLR2 [rs5743708A/G (Arg753Gln) and rs5743704A/C (Pro631-His)] polymorphisms were not associated with tuberculous meningitis (86). Whereas, a Chinese cohort study observed association of TLR2 rs3804099 T/C with susceptibility to tuberculous meningitis rather than with susceptibility to pulmonary TB (87). Another study conducted in Connecticut revealed the mechanism of TLR2 rs574370 (R753Q) polymorphism. The results suggested that R753Q polymorphism alters TLR2 signaling which leads to impaired MyD88-TLR2 assembly, downregulated IRAK-1 phosphorylation, diminished activation of MAPKs and NF-κB, and deficient induction of cytokines in macrophages infected with *M. Smegmatis* (88).

In Ghana study, screened exons of genes encoding TLR 1, 2, 4, and the adaptor molecule TIRAP. The results revealed a significant strong association of TLR1 “TT” genotype (rs3923647 A/T (His305Leu) with TB protection. This is supported by increased production of IFN-γ in BCG-stimulated PBMCs of “TT” genotype positive individuals (89). Another study screened the TLR and TIRAP gene markers to predict latent tuberculosis infection (LTBI) and active disease in a Chinese population. The analysis revealed the association of TLR2 “CC” genotype (rs3804100C/T) and TLR9 “TC” genotype (rs5743836) with LTBI susceptibility and TLR2 “GA” genotype (rs5743708A/G), TLR4 “GG” genotype (rs7873784C/G) and TLR8 “CC” genotype (rs3764879C/G) with susceptibility to pulmonary TB (90). In a Vietnamese cohort study in TLR9 polymorphism, rs352142G/T was associated with meningeal TB while rs352143A/G was associated with pulmonary TB (91). It has been reported that TLR4 rs4986790A/G (Asp299Gly) and rs4986791C/T (Thr399Ile) SNPs were associated with susceptibility to pulmonary TB in an Iranian population (92). Moreover, in Croatian population “AA” genotype of TLR10 SNP rs11096957A/C was found an increased risk with TB (93).

### Vitamin D receptor (VDR)

Vitamin D (Cholecalciferol) exerts its pleiotropic effects through the vitamin D receptor (VDR) which is present in the majority of the immune cells, including macrophages, B and T lymphocytes, neutrophils and DCs (94, 95). Vitamin D deficiency has been reported to be associated with TB susceptibility (96–98). Moreover, influence of vitamin D receptor gene variants on vitamin D modulated innate and adaptive immune response against Mtb has been reported. Association of VDR polymorphisms to TB susceptibility reported in several studies (99–101). In South Indian population reported that TaqI “tt” genotype associated with susceptibility to pulmonary TB in female patients, while BsmI “Bb” and FokI “FF” genotypes in male patients (102, 103). In the same population, the promoter polymorphisms Cdx2 G/A and A1012G revealed that genotype “GG” of Cdx2 associated with protection and haplotype A-A (A allele of Cdx-2 and A allele of A1012G) with susceptibility to pulmonary tuberculosis (104). Moreover, role in immune regulation of VDR gene variants also explored. It has been reported that Cdx2 “AA” and TaqI “tt” genotypes regulate the chemokine mediated inflammatory responses in T-cell subsets (105, 106). In addition, Cdx-2 “GG” genotype and “baT” haplotype are associated with a suppressed Th1 and increased IL-10 response and BsmI “BB,” TaqI “tt,” and the extended genotype “BBAAtt” shown to be associated with increased expression of VDR which in turn regulate the vitamin D_3_ modulated macrophage phagocytosis and lymphoproliferative immune functions to culture filtrate antigen of Mtb in healthy controls (107, 108). These studies suggested the anti-inflammatory effect of vitamin D which could be beneficial to avoid tissue injury and faster recovery during active TB disease. Whereas, in north Indian study TaqI “Tt” and “tt” genotypes associated with tuberculous meningitis compared with controls and pulmonary TB patients (87). In a Gujarati Indian population living in London, the *Fok*I ff genotype was strongly associated with PTB (109). In a study carried out in the Gambian PTB patients, the *Taq*I “tt” genotype was found less frequently in patients, suggesting that this genotype may be associated with resistance (110). A study from Turkey reported that the BsmI “B” allele was overrepresented in TB patients compared with healthy controls (111), whilst other studies have described prevalence of the “BB” genotype in TB (112). A meta-analysis study with 29 studies revealed that homozygote for the mutant allele of the *Apa*I polymorphism and heterozygote for the *Bsm*I polymorphism appeared to have a protective role on tuberculosis development in European populations. The same study also revealed that *Fok*I ff genotype is associated with a significantly increased risk of tuberculosis in Chinese population (113). In Iranian population no such association was found. However, vitamin D deficiency was strongly associated with TB susceptibility (114). In Han Taiwanese population, TaqI (rs731236), BsmI (rs1544410) and vitamin D binding protein (VDBP) polymorphisms were studied and reported that TaqI “AA,” BsmI “GG,” and VDBP “AA+AC” genotypes were found risk for development of TB (115). A meta-analysis carried out in 32 studies showed that FokI polymorphism associated with TB susceptibility in Asian population, in contrary, no association was found in Caucasian and African populations (100). Another meta-analysis of 16 studies revealed that FokI polymorphism associated with TB risk in East Asian population among different ethnicities. This indicates the association of FokI polymorphism with TB risk in Asia. In contrast, no association was found in TaqI, BsmI and ApaI polymorphisms among different ethnicities (101). Moreover a meta-analysis including 34 studies confirmed that in East and Southeast Asian populations, the homozygote model (ff vs. FF) and the recessive model (ff vs. Ff + FF) of FokI polymorphism associated with TB risk (116).

### Killer-cell immunoglobulin-like receptor (KIR)

Killer-cell immunoglobulin-like receptors are the regulatory molecules expressed on natural-killer (NK) cells. It mediates linking of innate and adaptive immune responses by the production of cytokines and modulate NK cell activity through stimulatory/inhibitory signals generated when ligands binds with KIR molecules on NK cells (117). KIR molecules are highly polymorphic and its diversity associated with TB outcome in different ethnic populations (118–120). It has been reported that the genotype AH and FZ14 may associated with Mtb clearance and haplotype B and haplotype A associated with susceptibility and protection with pulmonary TB (121). In Chinese Han population, 15 KIR activating and inhibitory genes were studied. The results shown that stimulatory genes such as *2DS1, 2DS3*, and *3DS1* associated with resistance to pulmonary tuberculosis (122). Similar protective association was observed in stimulatory *3DS1* gene in South African colored population (123). In Lur population of Iran investigated KIR as well as KIR-HLA combinations to TB susceptibility. They found a significant decrease in KIR3DS1 as well as KIR3DS1 with HLA-B Bw4IIe80 ligand frequencies in TB patients compared with controls. This suggested the combination of KIR3DS1+HLA-B Bw4LLe80 with susceptibility to TB in this population (124). Another study conducted in Iranian individuals on 17 KIR genes and their three major HLA class I ligand groups (-C1, -C2 and -Bw4: -B Bw4 (Ile80), -B Bw4 (Thr80) and -A Bw4) showed no significant association with pulmonary TB (125).

### Solute carrier family 11A member 1 (SLC11A1)

Solute carrier family 11A member 1 also known as natural resistance-associated macrophage protein 1 (NRAMP1) is a member of proton-coupled divalent metal ion transporters. Polymorphisms in this gene have been associated with tuberculosis, leprosy and other inflammatory diseases (126). Studies conducted in Japanese, Koreans, West Africans and South Africans reported that 3′untranslated region Asn543Asp (G to A at codon 543, resulting in an aspartic acid to asparagine change) and (TGTG) deletion in the 3′ untranslated region (1,729 + 55del4) (rs17235416) polymorphisms were associated with TB susceptibility (127–129). Moreover, in Gambian and Guineans intron 4 (469 + 14 G/C) (rs3731865) polymorphism and (CA)n repeat polymorphism in the immediate 5′ region among Gambians, Japanese, South Africans and Americans have also been reported to TB risk (128–132). The heterozygosity of CAAA insertion/deletion polymorphism of 3′UTR polymorphism associated with protection against TB in HIV positive and negative individuals in Karonga district of northern Malawi (133). Meta-analysis conducted in 14 case-control studies revealed that 3′UTR Asn543Asp (rs17235409) and 5′ (GT)n polymorphisms were associated with susceptibility to TB (134). Another meta-analysis in China studied3′UTR TGTG ins/del, D543N, INT4 (single nucleotide G to C change in intron 4) and (GT)n in the 5′ promoter region of *SLC11A1* gene. The analysis revealed that “A” allele carriers of D543N, TGTG ins/del, “C” allele carriers of INT4 and another allele carriers of (GT)n were associated with TB risk. Analysis stratified by ethnicity, subgroup of Asians found more susceptible to TB while an increased risk of extra-pulmonary TB was found for D543N polymorphism (135). Other studies in Chinese populations reported that deletion of TGTG in 3′UTR polymorphism TGTG+/del genotype associated with susceptibility to TB (53, 136). In contrary, studies conducted in Taiwanese, South Indian and Turkish population no significant association was found in TB (137–139).

### Cytokines and its receptors

Cytokines and chemokines are produced from various immune cells in response to external stimuli. They mediate its action through binding with specific receptors and modulate the immune functions. Polymorphisms in promoter as well as coding region of these genes may alter their transcriptional activation and its production. Studies have reported the association of altered cytokines/chemokines with susceptibility to infectious diseases including TB (140, 141).

### Interferon-gamma (IFN-γ)

IFN-γ is a Th1 type cytokine plays an important role in Mtb infections (142). In intron polymorphism of IFN-γ +874A/T, “T” allele and “TT” genotype associated with protection to tuberculosis in Sicilian and South African populations while associated with susceptibility in Pakistani population (143–145). In Spanish and Hong Kong Chinese population allele “A” and “AA” genotype associated with susceptibility to TB (146, 147). In contrary, no association was found in Turkish, Malawian, African American, Caucasian, Hispanics West African, South Indian and Chinese populations (148–153). Moreover, a meta-analysis study from Brazil reported the association of allele “T” with TB (141). In addition, polymorphisms in IFN-γ receptor genes have been reported in different ethnic population, but the results were inconsistent (154–159). Recently, 22 gene polymorphisms were studied in IFNGR1 and IFNGR2 gene among Korean TB patients, in which four IFNGR1 variants (rs9376269C/G, rs9376268A/G, rs9376267C/T, rs56251346C/T) were marginally associated with the risk of TB but not statistically significant (160). Another study analyzed 20 genes involved in IFN-γ signaling such as *IFNG, IFNGR1, IFNGR2, IRF1, IL12A, IL12B, IL12RB1, IL12RB2, IL23A, IL23R, IL27, EBI3, IL27RA, IL6ST, SOCS1, STAT1, STAT4, JAK2, TYK2*, and *TBX2*1 in a Ghanaian population but no significant result was observed in TB (20).

### Interleukin-12 (IL-12)

Interleukin-12 mainly involved in T-cells into Th1 cells and induce the production of INF-γ from T-cells and NK-cells. The intron 2 polymorphism +1188 of IL12B in south Indian population no significant association was observed (152, 161). Similarly, in African American and White ethnicities, no association was found in the 3′UTR polymorphism of IL12B (162). The study carried out in two West African populations and South and North Americans revealed the association of 3′UTR polymorphisms of IL12B with TB risk in African ancestry (163). In Morroccan population *IL12RB1* gene was studied which encodes beta 1 chain of the receptor for interleukin (IL)-12 and involved in Mendelian susceptibility to mycobacterial diseases. The study reported that two polymorphisms −2C → T and −111A → T were associated with TB risk (164). Another study reported that in haplotypes of *IL12RB1* gene “allele 2” R214-T365-R378 and “ATGG” haplotype of (641A/G, 1094T/C, 1132C/G, 1573G/A) associate with TB susceptibility in the Japanese population and this variation may diminish receptor response to IL-12 and IL-23 which may lead to downregulation of IFN-γ mediated immunity (165, 166). Among five SNPs studied in *IL12RB1* gene lack of association was found in Korean population (167).

### Tumor necrosis factor-α (TNF-α)

Tumor necrosis factor-α is an inflammatory cytokine which mediates the recruitment of immune cells at the site of infection for granuloma formation during Mtb infection. Among the various ethnic populations, the promoter gene polymorphisms of TNF-α [−238 (G/A),−308 (G/A),−376 (G/A),−592 (A/C) and−819 (C/T)] were not associated with TB risk in South Indian, Chinese and Cambodian populations (153, 168, 169). However, in Sicilian and Colombian populations protective associations were found in TNF-α −308 (G/A) and haplotype combination of −308A−238G polymorphisms (170, 171). In contrary, TNF-α [−308 (G/A)] variants associated with pulmonary TB susceptibility in Iranian population (92). In a meta-analysis study four TNF-α polymorphisms rs1800629G/A (−308G/A), rs1800630C/A (−863C/A), rs1799724 (−857C/T) and rs361525G/A (−238G/A) were analyzed among 18 studies. The results showed that in all study participants allele “G” and “GG” genotype of −308G/A and −238G/A polymorphisms were associated with pulmonary TB risk. When the analyses were performed among ethnicity −308G/A variant was associated with pulmonary TB in Asians, while −238G/A variant was associated with pulmonary TB in African individuals (172).

### IL-2

In South Indian population IL-2−330 T/G and +160 G/T polymorphisms were studied; the results reported that “TT” genotype associated with protection toward pulmonary TB (152). The similar protective effect was observed in allele “T” and “TT” genotype was observed in IL-2−330 T/G polymorphism in Russian study (173). Whereas, the haplotype combination of IL-2 330 G+160 G associated with protection with TB in Iranian population (174).

### IL-4

The promoter polymorphisms of IL-4−590T/C,−1098G/T, and−33C/T were studied in Iranian population and found no association to TB (174). In the 5′UTR variant IL4 rs2070874(C/T), “T” allele associated with two fold risk of TB in North Indians (175). In the South Indian study, the promoter IL-4-590C/T and VNTR polymorphisms were studied. The results revealed that−590 “T” allele associated with susceptibility and “CC” genotype with protection to pulmonary TB. The lack of association was observed in VNTR polymorphism (152, 176). The same promoter polymorphism of IL-4 allele “C” and “CC, “CT” genotypes associated with TB in Russian population (173).

### IL-6

In Colombian and South Indian populations no significant association was found in the IL-6 promoter −174(G/C) polymorphism (152, 177) while in Iranian and Canadian populations allele “G” and “GG” genotype associated with TB susceptibility (174, 178). Another study reported that the combinations of “TT-GG” of IFN-γ +874(T/A) and IL-6−174(G/C) polymorphisms associated with TB risk while combinations of “AA-GC” associated with protection to TB (179).

### IL-10

The promoter polymorphism of IL-10−1082(G/A) was studied in Cambodian and Sicilian populations, the results found that the allele “A” carriers was associated with TB risk, whereas in Turkish population the haplotype combinations “GCC” and “ACC” of IL-10−1,082 G/A,−819 C/T,−592 C/A polymorphisms were associated with susceptibility and protection to TB (169, 170, 180). In Colombian population “AA” genotype reported to be associated with pleural TB (177). In promoter IL-10−819C and−592C reported to be associated with TB risk in Turkish population (181). Another study reported that genotypes “GG” and “GA” of IL-10−1082 associated with TB (182). Moreover, the diplotype “AT/CC” of IL-10 (1800872/1800871) polymorphisms associated with TB risk in an Amerindian population (183). In contrary, no association was found in South Indian, Chinese, Turkish, Gambian and Spanish populations (152, 153, 161, 180, 184–186). Other candidate genes such as *chemokines, DC-SIGN (CD209)* and *Mannose binding lectin-2 (MBL-2)* associated with TB given in Tables 2–4.

**Table 2:** Association of DC-SIGN polymorphisms among different ethnic populations.

**DC-SIGN gene polymorphisms**	**Population/Country**	**Allele/haplotype**	**Association**	**Sample size**		**References**
				**HCs**	**TB**	
−871(A/G)−336(A/G)	South African	−871G −336A	Protection	360	351	(187)
−939(G/A)	Indonesian Vietnamese	GG vs. GA+AA	Protection No association			(188)
−336(A/G)	South Indian	−336GG	Susceptibility to PTB in HIV	157	107	(189)
	African	−336G	Protection	914	1,262	(190)
	Asian	−336GG	Susceptibility	3,610	3,539	(191)
	Asian	−336GG	Susceptibility	3,088	3,114	(192)
		−871G	Protection			
−336(A/G)	Spanish		No association	299	110	(193)
DC-SIGNR VNTR	Iranian		No association	161	171	(194)
−336(A/G)139(A/G)	West African		No association	347	321	(195)
−336(A/G)	Chinese		No association	2614	2598	(196)

*DC-SIGN, Dendritic Cell-Specific Intercellular adhesion molecule-3-Grabbing Non-integrin*.

**Table 3:** Chemokine gene polymorphisms in various ethnic populations.

**Chemokine gene polymorphisms**	**Population/Country**	**Allele/Haplotype**	**Association**	**Sample size**		**References**
				**HCs**	**PTB**	
**IL-8** −251 (T/A)	Whites/USA African American Paraguayan South Indian Gambian	−251“A”−251“T”	Susceptibility Protection No association	107 167 − 124 320	106 180 96 127 360	(197) (183) (198) (199)
**MCP-1 (CCL2)** −2518(G/A)	Mexican	“GG” and “AG”	Susceptibility	518	435	(200)
	Korean			162	129	
	Brazilian		No association	–	627	(201)
	Morroccan	−2518GG	Protection	–	–	(202)
−2362C	West African	2362C	Protection	2300	2000	(203)
**RANTES (CCL5)**−403(G/A)−28(C/G)In1.1 (T/C)	Chinese	A-C-T, G-C-C (Haplotype) Diplotypes (-403&In1.1) GA/TT; GG/TC	Susceptibility	465	412	(204)
	Tunisian	−28GG G-G, A-C (Haplotypes) AG/GC (diplotypes) Of-403&-28		150	168	(205)
	South Indian	A-C-C (haplotype) G/A-T/C (diplotype)	Protection	213	212	(206)
	North Indian	−403G/A	Susceptibility	215	215	(207)
	Sudanese	−28G		206	191	(208)
**MIP-1**α **(CCL3)** −459(C/T)	Mexican Korean		No association	518	435	(200)
**MIP-1**β **(CCL4)** Rs1634514(T/A) Rs1719144(G/A)	Brazilian	rs1634514T rs1719144A	Susceptibility	–	627	(201)
**CCL18** rs2015086(T/C) rs2015070(G/A) rs14304(G/A)	Brazilian	rs2015086C rs2015070A rs14304A	Protective	–	627	(201)
**IP-10 (CXCL10)**−135(G/A)−1447(A/G) −872(G/A)	Chinese		Susceptibility No association	176	240	(209)

**Table 4:** Association of MBL gene polymorphisms in different ethnic populations to TB.

**MBL2**	**Population/Country**	**Allele/Haplotype**	**Association**	**Sample size**		**References**
				**HCs**	**PTB**	
Exon1 (Codon 52, 54 and 57)	South Indian	Mutant homozygous	Susceptibility	109	202	(210)
Exon 1, codon-52	Chinese	Heterozygous	Susceptibility	198	125	(211)
−221Y/X	South Indian	O/O genotype YA/YA diplotype	Susceptibility Susceptibility to TB in HIV	146	148	(212)
Codon 54, 57	Turkish	AB genotype	Protection	99	27	(213)
Exon 1 −550(H/L) −221(X/Y) +4(P/Q)	Italian	HYA-HYA (Haplotype) LYB-LYD (Haplotype)	Protection Susceptibility	288	277	(214)
Exon 1 Codon54(A/B)	Indian	B' allele	Protection	392	286	(215)
−221(Y/X) Exon 1 Codon57(A/C)	Brazilian	X/Y genotype ‘O' allele ‘C' allele and ‘AC' genotype	Susceptibility Susceptibility Susceptibility	148	155	(216)
Exon1 codon 54(A/B) +4 (P/Q)	Chinese	54 ‘B' allele +4 ‘P/Q'	Susceptibility No association	1190	1106	(217)
rs7096206(C/G) rs6695096(T/C) rs2273346(T/C)	Chinese	‘CG' genotype ‘TC' genotype ‘TC' genotype	Susceptibility Susceptibility Susceptibility	216 419	205 503	(218)(219)
−550 (H/L) Codon 52 (A/B) +4 (P/Q) −221 (X/Y)	Chinese	HL genotype HPYA, LPXA, LQYA & LPYB (Haplotypes)	Protection Susceptibility	453	151	(136)
Exon 1 (Codon 54 and 57)	Turkish		No association	50	50	(139)(220)
Exon1 Codon 52,54 and 57	West Africa		No association	2346	2010	(221)

*MBL, Mannose binding lectin*.

### Mannose receptor (CD206)

Mannose receptor or MRC1 is a type I transmembrane C-type lectin expressed on macrophages and dendritic cells. It recognizes ligands such as mannose-N-acetylglucosamine- and fucose-terminated glycoconjugates which includes lipoglycan and mannose-capped lipoarabinomannan (ManLAM). The binding of ManLAM leads to stimulation of a nuclear receptor peroxisome proliferator-activated receptor gamma (PPAR-γ) and triggers anti-inflammatory immune response (222). Polymorphisms may affect the expression level and its structure and function which may lead to susceptibility to pulmonary tuberculosis. In Chinese population, six SNPs (G1186A, G1195A, T1212C, C1221G, C1303T, and C1323T) in exon 7 of the MRC1 gene was studied. Genotypic analysis revealed that the AG genotype of G1186A (rs34039386) polymorphism was significantly correlated with pulmonary tuberculosis (223). Another study investigated SNPS in exon 7 of MRC1 gene in 595 Chinese Uygur and 513 Kazak subjects. In the Uygur, the frequency of allele G (*P* = 0.031, *OR* = 1.29, 95% *CI* = 1.02−1.62) and AA genotype (*P* = 0.033, *OR* = 1.64, 95% *CI* = 1.04–2.60) for G1186A was lower in the pulmonary TB than healthy control and associated with pulmonary TB (224). Recent studies carried out in four functional MBL gene polymorphisms (HL, XY, PQ, and AB) found that individuals carrying BB/AB genotypes were associated with increased susceptibility to TB. Moreover, LYPB haplotype showed a significant association with increased risk of TB among Chinese population (225). Whereas, in Iranian Lur population MBL H allele and HH genotype significantly associated with increased susceptibility to TB (226).

### IL-17

IL-17 is a potent pro-inflammatory cytokine produced by activated T-lymphocytes and has a critical role in the immunopathology of TB. During primary Mtb infection, IL-17 induces the expression of chemokines that promote cell recruitment and granuloma formation. A balance between Th1 and Th17 responses is essential to control bacterial growth and limit immunopathology. The response toward excessive IL-17 production may cause excessive neutrophil recruitment and tissue damage. Thus, regulated immune response is essential to promote anti-mycobacterial immunity and prevent excessive immunopathological consequences (227). In china, a meta-analysis carried out for IL-17A rs22275913, rs3748067 and rs3819024; and IL-17F rs763780 polymorphisms in case-control studies published up to July 2017 involving 4,961 TB patients and 5,435 healthy controls. The results revealed that the rs2275913 polymorphism was associated with decreased TB risk in Caucasians under the allelic model (A vs. G, OR 0.69, 95%CI 0.49–0.96; *p* = 0.03), heterogeneous model (AG vs. GG, OR 0.67, 95%CI 0.47–0.93; *p* = 0.02) and domain model (AA+AG vs. GG, OR 0.64, 95%CI 0.44–0.93; *p* = 0.02). Whereas, rs3748067 was associated with increased TB risk in Asians under the homogeneous model (TT vs. CC, OR 1.36, 95%CI 1.03–1.79; *p* = 0.03) and recessive model (TT vs. CT+CC, OR 1.35, 95%CI 1.02–1.78; *p* = 0.03) (228).

## Genome-wide association studies

Genome-wide association studies (GWAS) is a hypothesis-free methodology which powered to identify variants that have a frequency of >5%. Five GWAS studies reported earlier, first study conducted in the Gambia and Ghana in 3,632 pulmonary TB patients and 7,501 healthy controls and confirmed that polymorphism in rs4331426 in Chr18q 11.2 was associated with pulmonary TB (5). Further, in Indonesian and Russian cohorts study identified the second variant associated with TB in rs2057178 on Chr11p13 in 13,859 PTB and 8,821 controls (6). The similar Chr11p13 variant association was studied in South African colored population, but not identified any loci associated with TB (229). The Russian study also identified a TB association in 11 SNPs in *ASAP1* gene in Chr8q24of5, 530 cases and 5,607 controls. Among the 11 SNPs, seven were more significant association with TB while these were found less common in Ghanaian and Gambian populations compared with Russian population. Moreover, this study demonstrated that “A” allele of SNP rs10956514 (A/G) alter ASAP1 levels in dendritic cells (230). In a replication study conducted in 1115 western Chinese Han and 914 Tibetan population, no association was found in ASAP1 rs10956514 (A/G) to TB risk (231). Family-based association tests (FBATs) performed in Morrocco revealed significant association of 143 SNPs. Among them four SNPs were significantly associated with pulmonary TB during the replication phase with 317 TB and 650 healthy controls. These SNPs are rs916943C/T in the *AGMO* (alkylglycerol monooxygenase) gene, rs358793A/G located near the *WNT5A* (Wingless-Type MMTV Integration Site Family, Member 5A) gene, rs17590261C/T located near the *PCDH10* (Protocadherin 10) gene and rs6786408A/C in the *FOXP1* (ForkheadBox P1) gene TB (232). Moreover, this study confirmed the association of SNPs (rs2057178C/T, rs11031731A/G, rs4331426 A/G) with TB (5, 6, 230, 232). The biological significance of most of the identified SNPs are unknown, however *AGMO* rs916943 C/T and *FOXP1* rs6786408 A/C were involved in macrophage functions (232). In Uganda and Tanzania, GWAS was conducted in 267 HIV-positive individuals who developed active TB and 314 HIV patients without active TB symptoms. The study identified one SNP in rs4921437 C/T which located 51 kb downstream from 3′UTR of *IL-12B* gene and found to be associated with protection to TB (233). Further analysis reported that this SNP was in strong linkage disequilibrium (LD) with rs10515780 C/G variant and both are located in the intron of ubiquitin-like-domain-containing C-terminal domain phosphatase 1 gene (*UBLCP1*) and in an H3K27Ac histone active enhancer which are involved in the regulatory mechanism (233). Recent genome-wide association study (GWAS) of 6,465 *Mycobacterium tuberculosis* clinical isolates from more than 30 countries characterized genetic determinants of resistance to anti-tuberculosis drugs. This study identifies high-quality genome wide SNPs, indels and large deletions across all samples. Phenotypic analysis revealed that 31.2% of isolates were resistant to at least one drug, with 15.1% categorized as MDR-TB and 4.3% categorized as XDR-TB. It also identified drug resistance loci such as *folC, ubiA, thyX*–*hsdS.1, thyA, alr, ald* and *dfrA*–*thyA*), new epistatic relationships, such as *pncA* with *pncB2* and *thyA* with *thyX*–*hsdS.1*) and efflux pumps represented by the ABC transporters *drrA* and *Rv2688c*. The novel genetic markers identified with resistance were SNPs in the *ethA* and *thyX* promoters. Besides SNPs, novels markers were found in small indels in *pncA* and *ald* and large deletions in prodrug activators such as *ethA* and *katG*. Most small indels were 1 bp in length and caused frameshifts and indels in *rpoB, pncA* and *embAB* promoter region were associated with MDR-TB and XDR-TB. It has been found that indels were distributed throughout the gene lengths; however, there was some evidence of areas of higher density, such as the *pncA* region between codons 130 and 132 and the codon 427–434 region in *rpoB*. In large deletions in *katG, ethA* and *pncA* genes activate prodrugs and none are essential to Mtb survival. These deletions occurred independently in different phylogenetic tree and are likely offer an alternative route to resistance as compared to SNPs, across lineages and populations. This implies that regional variation should be considered to fully characterize genotype–phenotype relationships. The associations identified may enlighten the molecular mechanisms underlying drug resistance and help in the development of novel antibiotics (234). Overall, GWASs identified certain loci associated with TB susceptibility and protection which needs to be confirmed with other ethnicities.

## Current genotyping methods

Newer sequencing methods such as pyro-sequencing, exome and whole-genome sequencing are able to detect rare variants with frequency < 1% to TB susceptibility and gives higher resolution of SNP genotyping. Whole exome sequencing, is a transcriptomics technique for sequencing all of the protein-coding genes in a genome known as the exomes which constituting about 1-2% of the human genome. This method is effective in the study of rare Mendelian diseases. The major advantage is to identify genetic variants that alter protein sequences at a much lower cost. A pilot study was conducted to evaluate exome sequencing to detect markers for TB susceptibility between active TB and latent TB. They identified 1,611 SNPs different from latent TB using the Sure Select kit while 182 SNPs were identified in TB not in latent TB individuals using TruSight kit (235). However, this study needs to be done in larger sample size. Whole genome sequencing method is useful to study the frequency of different mutations with Mendelian disorders in whole human genome. Similarly, RNA-sequencing method useful for enhanced resolution of transcripts and transcript variants, but the cost of RNA sequencing relatively high.

## Host genetics and tuberculosis treatment

Studies have studied the clinical impact of genetic factors in TB treatment. In china, anti-tuberculosis drug-induced hepatotoxicity (ATDH) was studied. The *in vivo* study shown to be associated with silent information regulator 1 (SIRT1), however the SNPs rs7069102C/G, rs2273773C/T, rs4746720C/T were not associated with ATDH (236). Some studies reported the bioavailability of TB drugs among different gene variants of Solute Carrier Organic Anion Transporter Family, Member 1B1 (SLCO1B1). Rifampicin activity depends on its concentration against Mtb. The study evaluated multidrug intensive-phase therapy in 72 adults with PTB from Africa, North America and Spain. The results were compared with 16 healthy controls from North America. Another study conducted in 60 TB patients of South African populations. Both the study results revealed that SNPs rs4149032C/T and rs11045819A/C were associated with lower concentrations and bio-availability of rifambicin (237, 238). In African 35 HIV positive TB patients, reduced rifabutin concentration was observed in SNPs rs4149032C/T, rs4149056C/T and rs2306283C/T, while 30% increased concentration of rifabutin was observed in heterozygous rs11045819 “AC” genotype compared to those with the homozygous rs11045819 “CC” genotype. The results suggested that SLCO1B1 polymorphisms may serve as a biomarker for the response to TB treatment (239). A GWAS conducted in Ethiopian patients with drug induced liver injury (DILI) and 354 anti-tuberculosis drug (ATD) tolerant patients and correlated with several SNPs. The replication phase was conducted in 27 DILI cases and 217 ATD tolerant individuals. The results shown association of the seven SNPs, such as rs10946737A/G, rs10946739C/T (close to FAM65B), rs320035A/G, rs393994C/T, rs320003A/G, rs319952C/T (close to AGBL4), and rs7958375A/G (close to CUX2) with ATD-induced liver toxicity (240). Polymorphisms in *CYP2E1*gene and its association with drug-induced liver disease (DILI) were studied in Indonesian TB patients. The results observed the association of rs3813867 “GG” genotype with an increased risk of DILI incidence (241). Polymorphism of NAT2 is associated with large inter-individual and inter-racial differences in the toxicity and efficacy of isoniazid. A study reported the safety and efficacy of a pharmacogenetics-based standard TB therapy for latent TB infection (LTBI) using NAT2 genotyping. This polymorphism shown to confer reduced acetylation of isoniazid, which results in higher drug levels and stratifying therapy based on NAT2 status reduces adverse outcomes (242). These studies suggested that host genetics play an important role in TB therapy and newer approaches needs to be emerge like host-directed immune-therapy, which have the potential to shorten the TB treatment, prevent resistance by promoting autophagy, antimicrobial peptide production by macrophages and other effector mechanisms. Several agents are in evaluation in phase II trials that could improve TB treatment outcome (243).

## Conclusion

Genetic factors involved in TB pathogenesis are polygenic, these factors may be helpful to identify disease markers for development or predisposition to TB. Overall, studies suggested that genetic markers identified in one population not consistent with other population due to ethnic variation. Moreover, these controversial results may also due to sample size and large degree of heterogeneity in study design. In addition, the biological functions of most of the identified markers using different methodologies have remained unknown. Hence, well designed work with large sample size and improved methodologies like exome sequencing, pyrosequencing and next generation sequencing (NGS) may provide high resolution information needed to provide a consistent genetic marker for TB disease. Moreover, genetic factors may play an important role in TB therapy and newer approaches needs to emerge to shorten the TB treatment.

## Author contributions

MH contributed to the review of literature, drafting and revising the manuscript, concept and design. PS and RB contributed to conception of idea, design and revision of the manuscript.

### Conflict of interest statement

The authors declare that the research was conducted in the absence of any commercial or financial relationships that could be construed as a potential conflict of interest.

## References

[1] WHO. Global Tuberculosis Report. Geneva: WHO (2017).

[2] Hill AV. Aspects of genetic susceptibility to human infectious diseases. Annu Rev Genet. (2006) 40:469–86. 10.1146/annurev.genet.40.110405.090546 17094741

[3] Bellamy R. Genome-wide approaches to identifying genetic factors in host susceptibility to tuberculosis. Microbes Infect. (2006) 8:1119–23. 10.1016/j.micinf.2005.10.025 16513396

[4] Takiff HE. Chapter 6: Host genetics and susceptibility. In: Palomino JC Leão SC Ritacco V editors. Tuberculosis 2007. From Basic Science to Patient Care. Tuberculosis Textbook.Com, 1st edn. Belgium: Flying Publisher (2007). pp. 207–62.

[5] Thye T Vannberg FO Wong SH Owusu-Dabo E Osei I Gyapong J. Genome-wide association analyses identifies a susceptibility locus for tuberculosis on chromosome 18q11.2. Nat Genet. (2010) 42:739–41. 10.1038/ng.639 20694014 PMC4975513

[6] Thye T Owusu-Dabo E Vannberg FO van Crevel R Curtis J Sahiratmadja E. Common variants at 11p13 are associated with susceptibility to tuberculosis. Nat Genet. (2012) 44:257–59. 10.1038/ng.1080 22306650 PMC3427019

[7] Mahasirimongkol S Yanai H Mushiroda T Promphittayarat W Wattanapokayakit S Phromjai J. Genome-wide association studies of tuberculosis in Asians identify distinct at-risk locus for young tuberculosis. J Hum Genet. (2012) 57:363–67. 10.1038/jhg.2012.35 22551897

[8] van Tong H Velavan TP Thye T Meyer CG. Human genetic factors in tuberculosis: an update. Trop Med Int Health (2017) 22:1063–71. 10.1111/tmi.12923 28685916

[9] Kallmann FJ Reisner D. Twin studies on the significance of genetic factors in tuberculosis. Am Rev Tuberc (1942) 47:549–74.

[10] Cooke GS Hill AV. Genetics of susceptibility to human infectious disease. Nat Rev Genet. (2001) 2:967–77. 10.1038/35103577 11733749

[11] Jouanguy E Altare F Lamhamedi S Revy P Emile JF Newport M. Interferon-gamma-receptor deficiency in an infant with fatal bacille Calmette-Guerin infection. N Engl J Med. (1996) 335:1956–61. 10.1056/NEJM199612263352604 8960475

[12] Newport MJ Huxley CM Huston S Hawrylowicz CM Oostra BA Williamson R. A mutation in the interferon-gamma-receptor gene and susceptibility to mycobacterial infection. N Engl J Med. (1996) 335:1941–49. 10.1056/NEJM199612263352602 8960473

[13] Filipe-Santos O Bustamante J Chapgier A Vogt G de Beaucoudrey L Feinberg J. Inborn errors of IL-12/23- and IFN-gamma- mediated immunity: molecular, cellular, and clinical features. Semin Immunol. (2006) 18:347–61. 10.1016/j.smim.2006.07.010 16997570

[14] de Beaucoudrey L Samarina A Bustamante J Cobat A Boisson-Dupuis S Feinberg J. Revisiting human IL-12Rbeta1 deficiency: a survey of 141 patients from 30 countries. Medicine (Baltimore) (2010) 89:381–402. 10.1097/MD.0b013e3181fdd832 21057261 PMC3129625

[15] Prando C Samarina A Bustamante J Boisson-Dupuis S Cobat A Picard C. Inherited IL-12p40 deficiency: genetic, immunologic, and clinical features of 49 patients from 30 kindreds. Medicine (Baltimore) (2013) 92:109–22. 10.1097/MD.0b013e31828a01f9 23429356 PMC3822760

[16] Hambleton S Salem S Bustamante J Bigley V Boisson-Dupuis S Azevedo J. IRF8 mutations and human dendritic-cell immunodeficiency. N Engl J Med. (2011) 365:127–38. 10.1056/NEJMoa1100066 21524210 PMC3136554

[17] Bogunovic D Byun M Durfee LA Abhyankar A Sanal O Mansouri D. Mycobacterial disease and impaired IFN-gamma immunity in humans with inherited ISG15 deficiency. Science (2012) 337:1684–88. 10.1126/science.1224026 22859821 PMC3507439

[18] Filipe-Santos O Bustamante J Haverkamp MH Vinolo E Ku CL Puel A. X-linked susceptibility to mycobacteria is caused by mutations in NEMO impairing CD40-dependent IL-12 production. J Exp Med. (2006) 203:1745–59. 10.1084/jem.20060085 16818673 PMC2118353

[19] Bustamante J Arias AA Vogt G Picard C Galicia LB Prando C. Germline CYBB mutations that selectively affect macrophages in kindreds with X-linked predisposition to tuberculous mycobacterial disease. Nat Immunol. (2011) 12:213–21. 10.1038/ni.1992 21278736 PMC3097900

[20] Meyer CG Intemann CD Förster B Owusu-Dabo E Franke A Horstmann RD. No significant impact of IFN-γ pathway gene variants on tuberculosis susceptibility in a West African population. Eur J Hum Genet. (2016) 24:748–55. 10.1038/ejhg.2015.172 26242990 PMC4930082

[21] Chen BF Wang R Chen YJ Zhu Y Ding L Wen YF. Association between HLA-DRB1 alleles and tuberculosis: a meta-analysis. Genet Mol Res (2015) 14:15859–68. 10.4238/2015.December.1.37 26634553

[22] Tong X Chen L Liu S Yan Z Peng S Zhang Y. Polymorphisms in HLA-DRB1 gene and the risk of tuberculosis: a meta-analysis of 31 studies. Lung (2015) 193:309–18. 10.1007/s00408-015-9692-z 25787085

[23] Wamala D Buteme HK Kirimunda S Kallenius G Joloba M. Association between human leukocyte antigen class II and pulmonary tuberculosis due to mycobacterium tuberculosis in Uganda. BMC Infect Dis. (2016) 16:23. 10.1186/s12879-016-1346-0 26803588 PMC4724396

[24] Souza de Lima D Morishi Ogusku M Porto Dos Santos M de Melo Silva C M Alves de Almeida V. Alleles of HLADRB1^*^04 Associated with Pulmonary Tuberculosis in Amazon BrazilianPopulation. PLoS ONE (2016) 11:e0147543. 10.1371/journal.pone.0147543 26901036 PMC4764689

[25] Toyo-Oka L Mahasirimongkol S Yanai H Mushiroda T Wattanapokayakit S Wichukchinda N. Strain-based *HLA* association analysis identified HLA-DRB1^*^09:01 associated with modern strain tuberculosis. HLA (2017) 90:149–56. 10.1111/tan.13070 28612994

[26] Brahmajothi V Pitchappan RM Kakkanaiah VN Sashidhar M Rajaram K Ramu S. Association of pulmonary tuberculosis and HLA in South India. Tubercle (1991) 72:123–32. 10.1016/0041-3879(91)90039-U 1949215

[27] Jagannathan L Chaturvedi M Satish B Satish KS Desai A Subbakrishna DK. HLA-B^*^57 and gender influence the occurrence of tuberculosis in HIV infected people of south India. Clin Dev Immunol. (2011) 2011:549023. 10.1155/2011/549023 21876708 PMC3162975

[28] Ravikumar M Dheenadhayalan V Rajaram K Lakshmi SS Kumaran PP Paramasivan CN. Associations of HLA-DRB1, DQB1 and DPB1 alleles with pulmonary tuberculosis in south India. Tuber Lung Dis. (1999) 79:309–17. 10.1054/tuld.1999.0213 10707259

[29] Mehra NK Rajalingam R Mitra DK Taneja V Giphart MJ. Variants of HLADR2/DR51 group haplotypes and susceptibility to tuberculoid leprosy and pulmonary tuberculosis in Asian Indians. Int J Lepr Other Mycobact Dis. (1995) 63:241–8. 7602219

[30] Rajalingam R Mehra NK Jain RC Myneedu VP Pande JN. Polymerase chain reaction-based sequence-specific oligonucleotide hybridization analysis ofHLAclass II antigens in pulmonary tuberculosis: relevance to chemotherapy and disease severity. J Infect Dis. (1996) 173:669–76. 10.1093/infdis/173.3.669 8627031

[31] Rajalingam R Mehra NK Mehra RD Neolia S Jain RC Pande JN. HLA class I profile in Asian Indian patients with pulmonary tuberculosis. Indian J Exp Biol. (1997) 35:1055–9. 9475039

[32] Sharma SK Turaga KK Balamurugan A Saha PK Pandey RM Jain NK.. Clinical and genetic risk factors for the development of multi-drug resistant tuberculosis in non-HIV infected patients at a tertiary care center in India: a case-control study. Infect Genet Evol. (2003) 3:183–8. 10.1016/S1567-1348(03)00086-8 14522182

[33] Singh SP Mehra NK Dingley HB Pande JN Vaidya M. C. HLA-A, -B, -C and –DR antigen profile in pulmonary tuberculosis in North India. Tissue Antigens (1983) 21:380–84. 10.1111/j.1399-0039.1983.tb00187.x 6868057

[34] Singh SP Mehra NK Dingley HB Pande JN Vaidya MC. Human leukocyte antigen (HLA)-linked control of susceptibility to pulmonary tuberculosis and association with HLA-DR types. J Infect Dis. (1983) 148:676–81. 10.1093/infdis/148.4.676 6415179

[35] Balamurugan A Sharma SK Mehra NK. Human leukocyte antigen class I supertypes influence susceptibility and severity of tuberculosis. J Infect Dis. (2004) 189:805–11. 10.1086/381689 14976596

[36] Mishra G Kumar N Kaur G Jain S Tiwari P K Mehra N K. Distribution of HLA-A, B and DRB1 alleles in Sahariya tribe of North Central India: an association with pulmonary tuberculosis. Infect Genet Evol. (2014) 22:175–82. 10.1016/j.meegid.2013.08.019 23994122

[37] Selvaraj P Uma H Reetha AM Kurian SM Xavier T Prabhakar R. HLA antigen profile in pulmonary tuberculosis patients & their spouses. Indian J Med Res. (1998) 107:155–8. 9604542

[38] Sriram U Selvaraj P Kurian SM Reetha AM Narayanan PR. HLA-DR2 subtypes & immune responses in pulmonary tuberculosis. Indian J Med Res. (2001) 113:117–24. 11558319

[39] Rajeswari DN Selvaraj P Raghavan S Jawahar MS Narayanan PR. Influence of HLA-DR2 on perforin-positive cells in pulmonary tuberculosis. Int J Immunogenet. (2007) 34:379–84. 10.1111/j.1744-313X.2007.00703.x 17845310

[40] Selvaraj P Raghavan S Swaminathan S Alagarasu K Narendran G Narayanan PR. HLA-DQB1 and -DPB1 allele profile in HIV infected patients with and without pulmonary tuberculosis of south India. Infect Genet Evol (2008) 8:664–71. 10.1016/j.meegid.2008.06.005 18652916

[41] Selvaraj P Swaminathan S Alagarasu K Raghavan S Narendran G Narayanan P. Association of human leukocyte antigen-A11 with resistance and B40 and DR2 with susceptibility to HIV-1 infection in south India. J Acquir Immune Defic Syndr. (2006) 43:497–99. 10.1097/01.qai.0000233312.36226.76 17099315

[42] Raghavan S Selvaraj P Swaminathan S Narendran G. Short communication: association of HLA-A^*^1101 with resistance and B^*^4006 with susceptibility to HIV and HIV-TB: an in silico analysis of promiscuous T cell epitopes. AIDS Res Hum Retroviruses (2009) 25:1023–8. 10.1089/aid.2009.0022 19803716

[43] Biman S Ajay W Sobhana M Mahendra B Krishnakali S Ranjana WM. Analysis of HLA association among North Indian HIV-positive individuals co-infected with*Mycobacterium tuberculosis*. Lung India. (2015) 32:449–52. 10.4103/0970-2113.164166 26628757 PMC4586997

[44] Liu ZQ Song CX Hu XL. Study of HLA-DR/DQ genes associated with tuberculosis of the bone and joint. J Chin Antituberc Assoc. (2000) 22:130–33.

[45] Wang JH Song CX Wang SM. Association of HLADRB1 genes with pulmonary tuberculosis. Chin J Tuberc Respir Dis. (2001) 24:302–5. 11802982

[46] Zhao YL Duan-Mu HJ Song CX. Analysis of the association between HLA-DRB1, DQB1 gene and pulmonary tuberculosis complicated with diabetes mellitus. Chin J Tuberc Respir Dis. (2001) 24:75–9. 11802942

[47] Liu ZH Luo YL Zhou L Xu WH Feng DY Tan YJ. A study on the correlation between HLA-DR genes and susceptibility to pulmonary tuberculosis in a population of Han nationality from southern China. Zhonghua Jie He He Hu Xi Za Zhi. (2004) 27:390–3. 15256088

[48] Xu L Yang YZ Tan WG. Study on possible role of HLA-DR and HLA-DQ gene type and haplotype in incidence of pulmonary tuberculosis in Shenzhen of China. Public Med Forum Mag. (2006) 10:387–90.

[49] Shi GL Hu XL Rong CX. Association of the polymorphism of HLA-DRB gene and ACE gene with pulmonary tuberculosis. J Chin Antituberc Assoc. (2007) 29:327–29.

[50] Ye SH Liu ML He C. Study of HLA-DRB1 genes associated with pulmonary tuberculosis. Chin J Blood Transfus. (2007) 20:42–3.

[51] Wang Xi He Peng Liu YuCai Wu Fang Wang Ping Zhang Le. Correlation between the polymorphism of HLA-DRB1 gene and the susceptibility to pulmonary tuberculosis in population of Uygur Nationality in Xinjiang. Chin J Zoonoses (2010) 26:417–20.

[52] Li SZ Jiang YJ Xie BW. Correlation between polymorphisms of DRB1, -DQA1, and -DQB1 alleles and susceptibility to pulmonary tuberculosis in Tibetan population of China. J Third Mil Med Univ. (2011) 33:1254–57.

[53] Wu F Zhang W Zhang L Wu J Li C Meng Xi. NRAMP1, VDR, HLADRB1, and HLA-DQB1 gene polymorphisms in susceptibility to tuberculosis among the Chinese Kazakh Population: a case-control study. Biomed Res Int. (2013) 2013:484535. 10.1155/2013/484535 24024195 PMC3758880

[54] Kim HS Park MH Song EY Park H Kwon SY Han SK. Association of HLADR and HLA-DQ genes with susceptibility to pulmonary tuberculosis in Koreans: preliminary evidence of associations with drug resistance, disease severity, and disease recurrence. Hum Immunol. (2005) 66:1074–81. 10.1016/j.humimm.2005.08.242 16386650

[55] Goldfeld AE Delgado JC Thim S Bozon MV Uglialoro AM Turbay D. Association of an HLA-DQ allele with clinical tuberculosis. JAMA (1998) 279:226–28. 10.1001/jama.279.3.226 9438744

[56] Lombard Z Dalton DL Venter PA Williams RC Bornman L. Association of HLA-DR,-DQ, and vitamin D receptor alleles and haplotypes with tuberculosis in the Venda of South Africa. Hum Immunol. (2006) 67:643–54. 10.1016/j.humimm.2006.04.008 16916662

[57] Terán-Escandón D Terán-Ortiz L Camarena-Olvera A González-Avila G Vaca-Marín MA Granados J. Human leukocyte antigen-associated susceptibility to pulmonary tuberculosismolecular analysis of class II alleles by DNA amplification and oligonucleotide hybridization in Mexican patients. Chest (1999) 115:428–33. 10.1378/chest.115.2.428 10027443

[58] Amirzargar AA Yalda A Hajabolbaghi M Khosravi F Jabbari H Rezaei N. The association of HLA-DRB, DQA1, DQB1 alleles and haplotype frequency in Iranian patients with pulmonary tuberculosis. Int J Tuberc Lung D (2004) 8:1017–21. 15305487

[59] Mahmoudzadeh-Niknam H Khalili G Fadavi P. Allelic distribution of human leukocyte antigen in Iranian patients with pulmonary tuberculosis. Hum Immunol. (2003) 64:124–9. 10.1016/S0198-8859(02)00703-6 12507823

[60] Harfouch-Hammoud EI Daher NA. Susceptibility to and severity of tuberculosis is genetically controlled by human leukocyte antigens. Saudi Med J. (2008) 29:1625–29. 18998014

[61] Duarte R Carvalho C Pereira C Bettencourt A Carvalho A Villar M. HLA class II alleles as markers of tuberculosis susceptibility and resistance. Rev Port Pneumol. (2011) 17:15–9. 10.1016/S0873-2159(11)70005-8 21251479

[62] Magira EE Papasteriades C Kanterakis S Toubis M Roussos C Monos DS. HLA-A and HLA-DRB1 amino acid polymorphisms are associated with susceptibility and protection to pulmonary tuberculosis in a Greek population. Hum Immunol. (2012) 73:641–46. 10.1016/j.humimm.2012.03.008 22504415

[63] Souza CF Noguti EN Visentainer JE Cardoso RF Petzl-Erler ML Tsuneto LT. HLA and MICA genes in patients with tuberculosis in Brazil. Tissue Antigens. (2012) 79:58–63. 10.1111/j.1399-0039.2011.01789.x 22032421

[64] Selby R Barnard JM Buehler SK Crumley J Larsen B Marshall WH. Tuberculosis associated with HLA—B8, BfS in a Newfoundland community study. Tissue Antigens. (1978) 11:403–8. 10.1111/j.1399-0039.1978.tb01275.x 694904

[65] Linda AL Leigh AS Peter WN Andrew ML Jodie SB Leisel CM. HLA-A, B, DRB1, DQA1, DQB1 alleles and haplotype frequencies in Dene and Cree cohorts in Manitoba, Canada. Hum Immunol (2017) 78:401–11. 10.1016/j.humimm.2017.03.009 28359736

[66] Cox RA Arnold DR Cook D Lundberg DI. HLA phenotypes in Mexican Americans with tuberculosis. Am Rev Respir Dis. (1982) 126:653–5. 6957158 10.1164/arrd.1982.126.4.653

[67] Ruggiero G Cosentini E Zanzi D Sanna V Terrazzano G Matarese G. Allelic distribution of human leucocyte antigen in historical and recently diagnosed tuberculosis patients in Southern Italy. Immunology. (2004) 111:318–22. 10.1111/j.1365-2567.2004.01811.x 15009432 PMC1782420

[68] Bothamley GH Beck JS Schreuder GM D'Amaro J de Vries RR Kardjito T. Association of tuberculosis and *M. tuberculosis-specific antibody levels with HLA*. J Infect Dis. (1989) 159:549–55. 10.1093/infdis/159.3.549 2464654

[69] Dubaniewicz A Lewko B Moszkowska G Zamorska B Stepinski J. Molecular subtypes of the HLA-DR antigens in pulmonary tuberculosis. Int J Infect Dis. (2000) 4:129–33. 10.1016/S1201-9712(00)90073-0 11179915

[70] Dubaniewicz A Moszkowska G Szczerkowska Z Hoppe A. Analysis of DQB1 allele frequencies in pulmonary tuberculosis: preliminary report. Thorax (2003) 58:890–1. 10.1136/thorax.58.10.890 14514946 PMC1746481

[71] Dubaniewicz A Moszkowska G Szczerkowska Z. Frequency of DRB1-DQB1 two-locus haplotypes in tuberculosis: preliminary report. Tuberculosis (Edinb) (2005) 85:259–67. 10.1016/j.tube.2004.12.003 15958261

[72] Vejbaesya S Chierakul N Luangtrakool K Srinak D Stephens HA. Associations of HLA class II alleles with pulmonary tuberculosis in Thais. Eur J Immunogenet. (2002) 29:431–4. 10.1046/j.1365-2370.2002.00352.x 12358854

[73] Khomenko AG Litvinov VI Chukanova VP Pospelov LE. Tuberculosis in patients with various HLA phenotypes. Tubercle (1990) 71:187–92. 10.1016/0041-3879(90)90074-I 2238125

[74] Mohammed NA Qassem H Hassen F. Association between HLA-Class, I., and HLA-Class II Alleles and *Mycobacterium tuberculosis* infection in iraqi patients from baghdad City. Iran J Med Sci. (2014) 39(2 Suppl.):191–5. 24753642 PMC3993047

[75] Hoffmann JA Kafatos FC Janeway CA Ezekowitz RA. Phylogenetic perspectives in innate immunity. Science (1999) 284:1313–18. 10.1126/science.284.5418.1313 10334979

[76] Kopp EB Medzhitov R. The Toll-receptor family and control of innate immunity. Curr Opin Immunol. (1999) 11:13–8. 10.1016/S0952-7915(99)80003-X 10047546

[77] Schnare M Barton GM Holt AC Takeda K Akira S Medzhitov R. Toll-like receptors control activation of adaptive immune responses. Nat Immunol. (2001) 2:947–50. 10.1038/ni712 11547333

[78] Faridgohar M Nikoueinejad H. New findings of Toll-like receptors involved in *Mycobacterium tuberculosis* infection. Pathog Glob Health (2017) 11:256–64. 10.1080/20477724.2017.1351080 PMC5560203 28715935

[79] Brightbill HD Libraty DH Krutzik SR Yang RB Belisle JT Bleharski JR. Host defense mechanisms triggered by microbial lipoproteins through tolllike receptors. Science (1999) 285:732–6. 10.1126/science.285.5428.732 10426995

[80] Abel B Thieblemont N Quesniaux VJ Brown N Mpagi J Miyake K. Toll-like receptor 4 expression is required to control chronic *Mycobacterium tuberculosis* infection in mice. J Immunol. (2002) 169:3155–62. 10.4049/jimmunol.169.6.3155 12218133

[81] Netea MG Wijmenga C O'Neill LA. Genetic variation in Toll-like receptors and disease susceptibility. Nat Immunol. (2012) 13:535–42. 10.1038/ni.2284 22610250

[82] Zhang Y Jiang T Yang X Xue Y Wang C Liu J. Toll-like receptor−1,−2, and−6 polymorphisms and pulmonary tuberculosis susceptibility: a systematic review and meta-analysis. PLoS ONE 8:e63357. 10.1371/journal.pone.0063357 23691034 PMC3653945

[83] Salie M Daya M Lucas LA Warren RM van der Spuy GD van Helden PD. Association of toll-like receptors with susceptibility to tuberculosis suggests sex-specific effects of TLR8 polymorphisms. Infect Genet Evol. (2015) 34:221–29. 10.1016/j.meegid.2015.07.004 26160538

[84] Bukhari M Aslam MA Khan A Iram Q Akbar A Naz AG. TLR8 gene polymorphism and association in bacterial load in southern Punjab of Pakistan: an association study with pulmonary tuberculosis. Int J Immunogenet. (2015) 42:46–51. 10.1111/iji.12170 25572425

[85] Dittrich N Berrocal-Almanza LC Thada S Goyal S Slevogt H Sumanlatha G. Toll-like receptor 1 variations influence susceptibility and immune response to *Mycobacterium tuberculosis*. Tuberculosis (2015) 95:328–35. 10.1016/j.tube.2015.02.045 25857934

[86] Rizvi I Garg RK Jain A Malhotra HS Singh AK Prakash S. Vitamin D status, vitamin D receptor and toll like receptor-2 polymorphisms in tuberculous meningitis: a case-control study. Infection (2016) 44:633–40. 10.1007/s15010-016-0907-x. 27207494

[87] Zhao Y Bu H Hong K Yin H Zou YL Geng SJ. Genetic polymorphisms of CCL1 rs2072069 G/A and TLR2 rs3804099 T/C in pulmonary or meningeal tuberculosis patients. Int J Clin Exp Pathol. (2015) 8:12608–20. 26722451 PMC4680396

[88] Pattabiraman G Panchal R Medvedev AE. The R753Q polymorphism in Toll-like receptor 2 (TLR2) attenuates innate immune responses to mycobacteria and impairs MyD88 adapter recruitment to TLR2. J Biol Chem (2017) 292:10685–95. 10.1074/jbc.M117.784470 28442574 PMC5481573

[89] Meyer CG Reiling N Ehmen C Ruge G Owusu-Dabo E Horstmann RD. TLR1 variant H305L associated with protection from pulmonary tuberculosis. PLoS ONE (2016) 11: e0156046. 10.1371/journal.pone.0156046 27214039 PMC4877073

[90] Wu L Hu Y Li D Jiang W Xu B. Screening toll-like receptor markers to predict latent tuberculosis infection and subsequent tuberculosis disease in a Chinese population. BMC Med Genet (2015) 16:19. 10.1186/s12881-015-0166-1 25928077 PMC4421918

[91] Graustein AD Horne DJ Arentz M Bang ND Chau TT Thwaites GE. TLR9 gene region polymorphisms and susceptibility to tuberculosis in Vietnam. Tuberculosis (2015) 95:190–6. 10.1016/j.tube.2014.12.009 25616954 PMC4573533

[92] Jafari M Nasiri MR Sanaei R Anoosheh S Farnia P Sepanjnia A. The NRAMP1, VDR, TNF-alpha, ICAM1, TLR2 and TLR4 gene polymorphisms in Iranian patients with pulmonary tuberculosis: A case-control study. Infect Genet Evol. (2016) 39:92–8. 10.1016/j.meegid.2016.01.013 26774366

[93] Bulat-Kardum LJ Etokebe GE Lederer P Balen S Dembic Z. Genetic polymorphisms in the Toll-like receptor 10, interleukin (IL)17A and IL17F genes differently affect the risk for tuberculosis in Croatian population. Scand J Immunol. (2015) 82:63–9. 10.1111/sji.12300 25857634

[94] Provvedini DM Tsoukas CD Deftos LJ Manolagas SC. 1,25-dihydroxy 1,25(OH)2D3 receptors in human leukocytes. Science (1983) 221:1181–3. 6310748 10.1126/science.6310748

[95] Baeke F Korf H Overbergh L van Etten E Verstuyf A Gysemans C. Human T lymphocytes are direct targets of 1,25-dihydroxy1,25(OH)2D3 in the immune system. J Steroid Biochem Mol Biol. (2010) 121:221–7. 10.1016/j.jsbmb.2010.03.037 20302932

[96] Gibney KB MacGregor L Leder K Torresi J Marshall C Ebeling PR. Vitamin D deficiency is associated with tuberculosis and latent tuberculosis infection in immigrants from sub-Saharan Africa. Clin Infect Dis. (2008) 46:443–46. 10.1086/525268 18173355

[97] Nnoaham K.E, Clarke A. Low serum vitamin D levels and tuberculosis: a systematic review and meta-analysis. Int J Epidemiol. (2008) 37:113–19. 10.1093/ije/dym247 18245055

[98] Martineau AR Leandro AC Anderson ST Newton SM Wilkinson K A Nicol MP. Association between Gc genotype and susceptibility to TB is dependent on vitamin D status. Eur Respir J. (2010) 35:1106–12. 10.1183/09031936.00087009 19797128 PMC2864196

[99] Cao Y Wang X Cao Z Cheng X. Association of VitaminD receptor gene TaqI polymorphisms with tuberculosis susceptibility: a meta-analysis. Int J Clin Exp Med. (2015) 8:10187–203. 26309718 PMC4538126

[100] Huang L Liu C Liao G Yang X Tang X Chen J. Vitamin D receptor gene FokI polymorphism contributes to increasing the risk of tuberculosis: an update meta-analysis. Medicine (Baltimore) 94:e2256. 10.1097/MD.0000000000002256 26705207 PMC4697973

[101] Lee YH Song GG. Vitamin D receptor gene FokI, TaqI, BsmI, and ApaI polymorphisms and susceptibility to pulmonary tuberculosis: a meta-analysis. Genet Mol Res. (2015) 14:9118–29. 10.4238/2015.August.7.21 26345844

[102] Selvaraj P Narayanan PR Reetha A. M. Association of vitamin D receptor genotypes with the susceptibility to pulmonary tuberculosis in female patients & resistance in female contactsIndian. J Med Res. (2000) 111:172–9. 10943070

[103] Selvaraj P Chandra G Kurian SM Reetha AM Narayanan P. R. Association of vitaminD receptor gene variants of Bsm, I, ApaI and FokI polymorphisms with susceptibility or resistance to pulmonary tuberculosis. Curr Sci. (2003) 84:1564–8.

[104] Selvaraj P Alagarasu K Harishankar M Vidyarani M Narayanan PR. Regulatory region polymorphisms of vitamin D receptor gene in pulmonary tuberculosis patients and normal healthy subjects of south India. Int J Immunogenet. (2008) 35:251–4. 10.1111/j.1744-313X.2008.00764.x 18397302

[105] Harishankar M Selvaraj P. Regulatory role of Cdx-2 and Taq I polymorphism of vitamin D receptor gene on chemokine expression in pulmonary tuberculosis. Hum Immunol. (2016) 77:498–505. 10.1016/j.humimm.2016.04.008 27067904

[106] Harishankar M Selvaraj P. Influence of Cdx2 and TaqI gene variants on vitamin D3 modulated intracellular chemokine positive T-cell subsets in pulmonary tuberculosis. Clin Ther (2017) 39: 46–57. 10.1016/j.clinthera.2017.04.003 28476406

[107] Selvaraj P Vidyarani M Alagarasu K Prabhu Anand S Narayanan PR. Regulatory role of promoter and 3' UTR variants of vitamin D receptor gene on cytokine response in pulmonary tuberculosis. J Clin Immunol (2008) 28:306–13. 10.1007/s10875-007-9152-5 18231846

[108] Selvaraj P Chandra G Jawahar MS Rani MV Rajeshwari DN Narayanan P. R. Regulatory role of vitamin D receptor gene variants of Bsm, I, Apa, I, Taq, I, and Fok I polymorphisms on macrophage phagocytosis and lymphoproliferative response to mycobacterium tuberculosis antigen in pulmonary tuberculosis. J Clin Immunol. (2004) 24:523–32. 10.1023/B:JOCI.0000040923.07879.31 15359111

[109] Wilkinson RJ Llewelyn M Toossi Z Patel P Pasvol G Lalvani A. Influence of vitamin D deficiency and vitamin D receptor polymorphisms on tuberculosis among Gujarati Asians in west London: a case-control study. Lancet (2000) 355:618–21. 10696983 10.1016/S0140-6736(99)02301-6

[110] Bellamy R Ruwende C Corrah T McAdam KP Thursz M Whittle HC. Tuberculosis and chronic hepatitis B virus infection in Africans and variation in the vitamin D receptor gene. J Infect Dis. (1999) 179:721–24. 9952386 10.1086/314614

[111] Ates O Dolek B Dalyan L Musellim B Ongen G Topal-Sarikaya A. The association between BsmI variant of vitamin D receptor gene and susceptibility to tuberculosis. Mol Biol Rep. (2011) 38:2633–36. 10.1007/s11033-010-0404-8 21104028

[112] Gao L Tao Y Zhang L Jin Q. Vitamin D receptor genetic polymorphisms and tuberculosis: updated systematic review and meta-ana lysis. Int J Tuberc Lung Dis. (2010) 14:15–23. 20003690

[113] Chen C Liu Q Zhu L Yang H Lu W. Vitamin D receptor gene polymorphisms on the risk of tuberculosis,a meta-analysis of 29 case-control studies. PLoS ONE (2013) 8:e83843. 10.1371/journal.pone.0083843 24349552 PMC3862802

[114] Rashedi J Asgharzadeh M. Moaddab S. R. Sahebi L. Khalili M. Mazani M.. (2014). Vitamin d receptor gene polymorphism and vitamin d plasma concentration: correlation with susceptibility to tuberculosis. Adv Pharm Bull 4(Suppl. 2): 607–11. 10.5681/apb.2014.089 25671196 PMC4312412

[115] Lee SW Chuang TY Huang HH Liu CW Kao YH Wu LS. VDR and VDBP genes polymorphisms associated with susceptibility to tuberculosis in a Han Taiwanese population. J Microbiol Immunol Infect. (2016) 49:783–87. 10.1016/j.jmii.2015.12.008 26869016

[116] Cao Y Wang X Cao Z Cheng X. Vitamin D receptor gene FokI polymorphisms and tuberculosis susceptibility: a metaanalysis. Arch Med Sci. (2016) 12:1118–34. 10.1371/journal.pone.0140634 27695504 PMC5016579

[117] Martin MP Carrington M. KIR locus polymorphisms: genotyping and disease association analysis. Methods Mol Biol. (2008) 415:49–64. 10.1007/978-1-59745-570-1_3 18370147 PMC10763752

[118] Méndez A Granda H Meenagh A Contreras S Zavaleta R Mendoza MF. Study of KIR genes in tuberculosis patients. Tissue Antigens. (2006) 68:386–9. 17092251 10.1111/j.1399-0039.2006.00685.x

[119] Mahfouz R Halas H Hoteit R Saadeh M Shamseddeen W Charafeddine K. Study of KIR genes in Lebanese patients with tuberculosis. Int J Tuberc Lung Dis. (2011) 15:1688–91. 10.5588/ijtld.11.0138 22118180

[120] Braun K Larcombe L Orr P Nickerson P Wolfe J Sharma M. Killer immunoglobulin-like receptor (KIR) centromeric-AA haplotype is associated with ethnicity and tuberculosis disease in a Canadian First Nations cohort. PLoS ONE (2013) 8:e67842. 10.1371/journal.pone.0067842 23861818 PMC3701593

[121] Lu C Bai XL Deng YF Wang CY Fan G Shen YJ. Killer cell immunoglobulin-like receptor genotypes and haplotypes with susceptibility topulmonary tuberculosis infection. Clin Lab. (2014) 60:821–25. 24839826 10.7754/clin.lab.2013.130404

[122] Lu C Shen YJ Deng YF Wang CY Fan G Liu YQ. Association of killer cell immunoglobulin-like receptors with pulmonary tuberculosis in Chinese Han. Genet Mol Res. (2012) 11:1370–78. 10.4238/2012.May.15.7 22653583

[123] Salie M Daya M Möller M Hoal EG. Activating KIRs alter susceptibility to pulmonary tuberculosis in a South African population. Tuberculosis (Edinb) (2015) 95:817–21. 10.1016/j.tube.2015.09.003 26542219

[124] Shahsavar F Mousavi T Azargon A Entezami K. Association of KIR3DS1+HLA-B Bw4Ile80 combination with susceptibility to tuberculosis in Lur population of Iran. Iran J Immunol. (2012) 9:39–47. 22426166 10.22034/iji.2012.16855

[125] Tajik N Shah-hosseini A Mohammadi A Jafari M Nasiri M Radjabzadeh MF. Susceptibility to pulmonary tuberculosis in Iranian individuals is not affected by compound KIR/HLA genotype. Tissue Antigens (2012) 79:90–6. 10.1111/j.1399-0039.2011.01812.x 22128749

[126] Teras I Ryigas E. M Kazakova II Ranne KHP Trapido LE. [“Detection of Trichomonas in the bronchi, sputum and oral cavity in various lung diseases]”. Terapevticheskii Arkhiv (1980) 52:123–5. 7385031

[127] Ryu S Park YK Bai GH Kim SJ Park SN Kang S. 3′UTR polymorphisms in the NRAMP1 gene are associated with susceptibility to tuberculosis in Koreans. Int J Tuberc Lung Dis. (2000) 4:577–80. 10864190

[128] Bellamy R Ruwende C Corrah T McAdam KP Whittle HC Hill AV. Variations in the NRAMP1 gene and susceptibility to tuberculosis in West Africans. N Engl J Med. (1998) 338:640–44. 9486992 10.1056/NEJM199803053381002

[129] Hoal EG Lewis LA Jamieson SE Tanzer F Rossouw M Victor T. SLC11A1 (NRAMP1) but not SLC11A2 (NRAMP2) polymorphisms are associated with susceptibility to tuberculosis in a high-incidence community in South Africa. Int J Tuberc Lung Dis. (2004) 8:1464–71. 15636493

[130] Gao PS Fujishima S Mao XQ Remus N Kanda M Enomoto T. Genetic variants of NRAMP1 and active tuberculosis in Japanese populations. International tuberculosis genetics team. Clin Genet (2000) 58:74–6. 10945666 10.1034/j.1399-0004.2000.580113.x

[131] Ma X Dou S Wright JA Reich RA Teeter LD El Sahly HM. 5′ dinucleotide repeat polymorphism of NRAMP1 and susceptibility to tuberculosis among Caucasian patients in Houston, Texas. Int J Tuberc Lung Dis. (2002) 6:818–23. 12234138

[132] Awomoyi AA Marchant A Howson JM McAdam KP Blackwell JM Newport MJ. Interleukin-10, polymorphism in SLC11A1 (formerly NRAMP1), and susceptibility to tuberculosis. J Infect Dis. (2002) 186:1808–14. 12447767 10.1086/345920

[133] Fitness J Floyd S Warndorff DK Sichali L Malema S Crampin AC. Large-scale candidate gene study of tuberculosis susceptibility in the Karonga district of northern Malawi. Am J Trop Med Hyg. (2004) 71:341–9. 15381817

[134] Li HT Zhang TT Zhou YQ Huang QH Huang J. SLC11A1 (formerly NRAMP1) gene polymorphisms and tuberculosis susceptibility: a metaanalysis. Int J Tuberc Lung Dis. (2006) 10:3–12. 16466030

[135] Meilang Q Zhang Y Zhang J Zhao Y Tian C Huang J. Polymorphisms in the SLC11A1 gene and tuberculosis risk: a metaanalysis update. Int J Tuberc Lung Dis. (2012) 16:437–46. 10.5588/ijtld.10.0743 22326178

[136] Wu L Deng H Zheng Y Mansjö M Zheng X Hu Y. An association study of NRAMP1, VDR, MBL and their interaction with the susceptibility to tuberculosis in a Chinese population. Int J Infect Dis. (2015) 38:129–35. 10.1016/j.ijid.2015.08.003 26261060

[137] Liaw YS Tsai-Wu JJ Wu CH Hung CC Lee CN Yang PC. Variations in the NRAMP1 gene and susceptibility of tuberculosis in Taiwanese. Int J Tuberc Lung Dis. (2002) 6:454–60. 12019922

[138] Selvaraj P Chandra G Kurian SM Reetha AM Charles N Narayanan PR. (2011. NRAMPI gene polymorphism in pulmonary and spinal tuberculosis.Curr Sci (2002) 82:451–4.

[139] Solgun H. A. TaStemir D. Aksaray N. Inan I. Demirhan O. Polymorphisms in NRAMP1 and MBL2 genes and their relations with tuberculosis in Turkish children. Tuberk Toraks (2011) 59:48–53. 21554230 10.5578/tt.2385

[140] Bidwell J Keen L Gallagher G Kimberly R Huizinga T McDermott MF.. Cytokine gene polymorphism in human disease: on-line databases. Genes Immun. (1999) 1:3–19. 11197303 10.1038/sj.gene.6363645

[141] Pacheco AG Cardoso CC Moraes MO. IFNG +874T/A, IL10−1082G/A and TNF−308G/A polymorphisms in association with tuberculosis susceptibility: a meta-analysis study. Hum Genet (2008) 123:477–84. 10.1007/s00439-008-0497-5 18414898

[142] Flynn JL Chan J Triebold KJ Dalton DK Stewart TA Bloom BR. An essential role for interferon gamma in resistance to Mycobacterium tuberculosis infection. J Exp Med. (1993) 178:2249–54. 7504064 10.1084/jem.178.6.2249 PMC2191274

[143] Lio D Marino V Serauto A Gioia V Scola L Crivello A. Genotype frequencies of the +874T—>A single nucleotide polymorphism in the first intron of the interferon-gamma gene in a sample of Sicilian patients affected by tuberculosis. Eur J Immunogenet. (2002) 29:371–4. 12358843 10.1046/j.1365-2370.2002.00327.x

[144] Rossouw M Nel HJ Cooke GS van Helden PD Hoal EG. Association between tuberculosis and a polymorphic NFkappaB binding site in the interferon gamma gene. Lancet (2003) 361:1871–2. 12788577 10.1016/S0140-6736(03)13491-5

[145] Ansari A Talat N Jamil B Hasan Z Razzaki T Dawood G. Cytokine gene polymorphisms across tuberculosis clinical spectrum in Pakistani patients. PLoS ONE (2009) 4:e4778. 10.1371/journal.pone.0004778 19274101 PMC2652824

[146] López-Maderuelo D Arnalich F Serantes R González A Codoceo R Madero R. Interferongamma and interleukin-10 gene polymorphisms in pulmonary tuberculosis. Am J Respir Crit Care Med. (2003) 167:970–75. 12531774 10.1164/rccm.200205-438BC

[147] Tso HW Ip WK Chong WP Tam CM Chiang AK Lau YL. Association of interferon gamma and interleukin 10 genes with tuberculosis in Hong Kong Chinese. Genes Immun. (2005) 6:358–63. 15815688 10.1038/sj.gene.6364189

[148] Oral HB Budak F Uzaslan EK Baştürk B Bekar A Akalin H. Interleukin-10 (IL-10) gene polymorphism as a potential host susceptibility factor in tuberculosis. Cytokine. (2006) 35:143–7. 16962335 10.1016/j.cyto.2006.07.015

[149] Fitness J Floyd S Warndorff DK Sichali L Malema S Crampin AC. Large-scale candidate gene study of tuberculosis susceptibility in the Karonga district of northern Malawi. Am J Trop Med Hyg. (2004) 71:341–9. 15381817

[150] Moran A Ma X Reich RA Graviss EA. No association between the +874T/A single nucleotide polymorphism in the IFN-gamma gene and susceptibility to tuberculosis. Int J Tuberc Lung Dis. (2007) 11:113–15. 17217140

[151] Cooke GS Campbell SJ Sillah J Gustafson P Bah B Sirugo G. Polymorphism within the interferon-gamma/receptor complex is associated with pulmonary tuberculosis. Am J Respir Crit Care Med. (2006) 174:339–43. 16690980 10.1164/rccm.200601-088OC

[152] Selvaraj P Alagarasu K Harishankar M Vidyarani M Nisha Rajeswari D Narayanan PR. *Cytokine* gene polymorphisms and cytokine levels in pulmonary tuberculosis. Cytokine. (2008) 43:26–33. 10.1016/j.cyto.2008.04.011 18522869

[153] Wu F Qu Y Tang Y Cao D Sun P Xia Z. Lack of association between cytokine gene polymorphisms and silicosis and pulmonary tuberculosis in Chinese iron miners. J Occup Health. (2008) 50:445–54. 18931463 10.1539/joh.l8006

[154] Fraser DA Bulat-Kardum L Knezevic J Babarovic P Matakovic-Mileusnic N Dellacasagrande J. Interferon-gamma receptor-1 gene polymorphism in tuberculosis patients from Croatia. Scand J Immunol. (2003) 57:480–4. 12753505 10.1046/j.1365-3083.2003.01253.x

[155] Sahiratmadja E Baak-Pablo R de Visser AW Alisjahbana B Adnan I van Crevel R. Association of polymorphisms in IL-12/IFN-gamma pathway genes with susceptibility to pulmonary tuberculosis in Indonesia. Tuberculosis (Edinb) (2007) 87:303–11. 17392024 10.1016/j.tube.2007.02.001

[156] Awomoyi AA Nejentsev S Richardson A Hull J Koch O Podinovskaia M. No association between interferon-gamma receptor-1 gene polymorphism and pulmonary tuberculosis in a Gambian population sample. Thorax (2004) 59:291–4. 10.1136/thx.2003.013029 15047947 PMC1763823

[157] Mirsaeidi SM Houshmand M Tabarsi P Banoei MM Zargari L Amiri M. Lack of association between interferon-gamma receptor-1 polymorphism and pulmonary tuberculosis in Iranian population sample. J Infect. (2006) 52:374–7. 16233916 10.1016/j.jinf.2005.08.009

[158] Park GY Im YH Ahn CH Park JW Jeong SW Ahn JY. Functional and genetic assessment of IFN-gamma receptor in patients with clinical tuberculosis. Int J Tuberc Lung Dis. (2004) 8:1221–7. 15527154

[159] Rosenzweig SD Schäffer AA Ding L Sullivan R Enyedi B Yim JJ. Interferon-gamma receptor 1 promoter polymorphisms: population distribution and functional implications. Clin Immunol. (2004) 112:113–9. 15207788 10.1016/j.clim.2004.03.018

[160] Shin JG Park BL Kim LH Namgoong S Kim JO Chang HS. Association study of polymorphisms in interferon-gamma receptor genes with the risk of pulmonary tuberculosis. Mol Med Rep. (2015) 12:1568–78. 10.3892/mmr.2015.3544 25815589

[161] Prabhu Anand S Selvaraj P Jawahar MS Adhilakshmi AR Narayanan PR. Interleukin-12B & interleukin-10 gene polymorphisms in pulmonary tuberculosis. Indian J Med Res. (2007) 126:135–38. 17932439

[162] Ma X Reich RA Gonzalez O Pan X Fothergill AK Starke JR. No evidence for association between the polymorphism in the 3′ untranslated region of interleukin-12B and human susceptibility to tuberculosis. J Infect Dis. (2003) 188:1116–18. 14551880 10.1086/378674

[163] Morris G. A. Edwards D. R. Hill P. C. Wejse C. Bisseye C. Olesen R.. (2011). Interleukin 12B (IL12B) genetic variation and pulmonary tuberculosis: a study of cohorts from The Gambia, Guinea-Bissau, United States and Argentina. PLoS ONE 6:e16656 10.1371/journal.pone.0016656 21339808 PMC3037276

[164] Remus N El Baghdadi J Fieschi C Feinberg J Quintin T Chentoufi M. Association of IL12RB1 polymorphisms with pulmonary tuberculosis in adults in Morocco. J Infect Dis. (2004) 190:580–87. 15243935 10.1086/422534

[165] Akahoshi M Nakashima H Miyake K Inoue Y Shimizu S Tanaka Y. Influence of interleukin-12 receptor beta1 polymorphisms on tuberculosis. Hum Genet. (2003) 112:237–43. 12596048 10.1007/s00439-002-0873-5

[166] Kusuhara K Yamamoto K Okada K Mizuno Y Hara T. Association of IL12RB1 polymorphisms with susceptibility to and severity of tuberculosis in Japanese: a gene-based association analysis of 21 candidate genes. Int J Immunogenet (2007). 34:35–44. 10.1111/j.1744-313X.2007.00653.x 17284226

[167] Lee HW Lee H S Kim D Ko DS Han S K Shim YS. Lack of an association between interleukin-12 receptor beta1 polymorphisms and tuberculosis in Koreans. Respiration (2005). 72: 365–8. 16088278 10.1159/000086249

[168] Selvaraj P Sriram U Mathan Kurian S Reetha AM Narayanan PR. Tumour necrosis factor alpha (-238 and−308) and beta gene polymorphisms in pulmonary tuberculosis: haplotype analysis with HLA-A, B and DR genes. Tuberculosis (Edinb). (2001) 81:335–41. 11800584 10.1054/tube.2001.0307

[169] Delgado JC Baena A Thim S Goldfeld AE. Ethnic-specific genetic associations with pulmonary tuberculosis. J Infect Dis. (2002) 186:1463–8. 12404162 10.1086/344891

[170] Scola L Crivello A Marino V Gioia V Serauto A Candore G. IL-10 and TNF-alpha polymorphisms in a sample of Sicilian patients affected by tuberculosis: implication for ageing and life span expectancy. Mech Ageing Dev. (2003) 124:569–72. 12714269 10.1016/s0047-6374(03)00038-1

[171] Correa PA Gomez LM Cadena J Anaya JM. Autoimmunity and TB. Opposite association with TNF polymorphism J Rheumatol. (2005) 32:219–24. 15693080

[172] Yi YX Han JB Zhao L Fang Y Zhang YF Zhou GY. Tumor necrosis factor alpha gene polymorphism contributes to pulmonary tuberculosis susceptibility: evidence from a meta-analysis. Int J Clin Exp Med. (2015) 8:20690–700. 26884992 PMC4723837

[173] Naslednikova IO Urazova OI Voronkova OV Strelis AK Novitsky VV Nikulina EL. Allelic polymorphism of cytokine genes during pulmonary tuberculosis. Bull Exp Biol Med. (2009) 148:175–80 10.1007/s10517-009-0674-0 20027321

[174] Amirzargar AA Rezaei N Jabbari H Danesh AA Khosravi F Hajabdolbaghi M. Cytokine single nucleotide polymorphisms in Iranian patients with pulmonary tuberculosis. Eur Cytokine Netw. (2006) 17:84–9. 16840026

[175] Abhimanyu BM Jha P Indian Genome Variation Consortium. Footprints of genetic susceptibility to pulmonary tuberculosis: cytokine gene variants in north Indians. Indian J Med Res. (2012) 135:763–70. 22771610 PMC3401711

[176] Vidyarani M Selvaraj P Prabhu Anand S Jawahar MS Adhilakshmi AR Narayanan PR. Interferon gamma (IFNgamma) &interleukin-4 (IL-4) gene variants&cytokine levels in pulmonary tuberculosis. Indian J Med Res. (2006) 124:403–10. 17159260

[177] Henao MI Montes C París SC García LF. Cytokine gene polymorphisms in Colombian patients with different clinical presentations of tuberculosis. Tuberculosis (Edinb). (2006) 86:11–9. 15925543 10.1016/j.tube.2005.03.001

[178] Larcombe LA Orr PH Lodge AM Brown JS Dembinski IJ Milligan LC. Functional gene polymorphisms in canadian aboriginal populations with high rates of TB. J Infect Dis. (2008) 198:1175–9. 10.1086/592049 18713057

[179] Ansari A Hasan Z Dawood G Hussain R. Differential combination of cytokine and interferon- γ +874 T/A polymorphisms determines disease severity in pulmonary tuberculosis. PLoS ONE 6:e27848. 10.1371/journal.pone.0027848 22140472 PMC3226558

[180] Ates O Musellim B Ongen G Topal-Sarikaya A. Interleukin-10 and tumor necrosis factor-alpha gene polymorphisms in tuberculosis. J Clin Immunol. (2008) 28:232–6. 18071881 10.1007/s10875-007-9155-2

[181] Akgunes A Coban AY Durupinar B. Human leucocyte antigens and cytokine gene polymorphisms and tuberculosis. Indian J Med Microbiol. (2011). 29:28–32. 10.4103/0255-0857.76520 21304191

[182] Meenakshi P Ramya S Shruthi T Lavanya J Mohammed HH Mohammed SA. Association of IL-1β +3954 C/T and IL-10-1082 G/A cytokine gene polymorphisms with susceptibility to tuberculosis. Scand J Immunol.(2013) 78:92–7. 10.1111/sji.12055 23654353

[183] Lindenau JD Guimarães LS Friedrich DC Hurtado AM Hill KR Salzano FM. Cytokine gene polymorphisms are associated with susceptibility to tuberculosis in an Amerindian population. Int J Tuberc Lung Dis (2014) 18:952–57. 10.5588/ijtld.14.0060 25199010

[184] Bellamy R Ruwende C Corrah T McAdam KP Whittle HC Hill AV. Assessment of the interleukin 1 gene cluster and other candidate gene polymorphisms in host susceptibility to tuberculosis. Tuber Lung Dis. (1998) 79:83–9. 10645445 10.1054/tuld.1998.0009

[185] López-Maderuelo D Arnalich F Serantes R González A Codoceo R Madero R. Interferongamma and interleukin-10 gene polymorphisms in pulmonary tuberculosis. Am J Respir Crit Care Med. (2003) 167:970–5. 12531774 10.1164/rccm.200205-438BC

[186] Harishankar M Ravikrishnan H Ravishankar A Elizabeth LH Swaminathan S Selvaraj P. IL-10 promoter−592 polymorphism may influence resistance to HIV infection in South Indian population. Curr HIV Res. (2018) 16:58–63. 10.2174/1570162X16666180219153752 29468971

[187] Barreiro LB Neyrolles O Babb CL Tailleux L Quach H McElreavey K. Promoter variation in the DC-SIGN-encoding gene CD209 is associated with tuberculosis. PLoS Med (2006) 3:e20. 10.1371/journal.pmed.0030020 16379498 PMC1324949

[188] Kobayashi K Yuliwulandari R Yanai H Lien LT Hang NT Hijikata M. Association of CD209 polymorphisms with tuberculosis in an Indonesian population. Hum Immunol. (2011) 72:741–45. 10.1016/j.humimm.2011.04.004 21704100

[189] Selvaraj p Alagarasu K Swaminathan S Harishankar M Narendran G. CD209 gene polymorphisms in South Indian, HIV and HIV-TB patients. Infect Genet Evol (2009) 9:256–62. 10.1016/j.meegid.2008.12.003 19126442

[190] Vannberg FO Chapman SJ Khor CC Tosh K Floyd S Jackson-Sillah D. CD209 genetic polymorphism and tuberculosis disease. PLoS ONE (2008) 3:e1388. 10.1371/journal.pone.0001388 18167547 PMC2148105

[191] Chang K Deng S Lu W Wang F Jia S Li F. Association between CD209−336A/G and−871A/G polymorphisms and susceptibility of tuberculosis: a meta-analysis. PLoS ONE (2012) 7:e41519. 10.1371/journal.pone.0041519 22911807 PMC3404017

[192] Yi L Zhang K Mo Y Zhen G Zhao J. The association between CD209 gene polymorphisms and pulmonary tuberculosis susceptibility: a meta-analysis. Int J Clin Exp Pathol. (2015) 8:12437–45. 26722430 PMC4680375

[193] Gómez LM Anaya JM Sierra-Filardi E Cadena J Corbí A Martín J. Analysis of DC-SIGN (CD209) functional variants in patients with tuberculosis. Hum Immunol. (2006) 67:808–11. 17055357 10.1016/j.humimm.2006.07.003

[194] Naderi M Hashemi M Taheri M Pesarakli H Eskandari-Nasab E Bahari G. CD209 promoter−336 A/G (rs4804803) polymorphism is associated with susceptibility to pulmonary tuberculosis in Zahedan, southeast Iran. J Microbiol Immunol Infect. (2014) 47:171–75. 10.1016/j.jmii.2013.03.013 23751770

[195] Olesen R Wejse C Velez DR Bisseye C Sodemann M Aaby P. DC-SIGN (CD209), pentraxin 3 and vitamin D receptor gene variants associate with pulmonary tuberculosis risk in West Africans. Genes Immun. (2007) 8:456–67. 17611589 10.1038/sj.gene.6364410

[196] Miao R Li J Sun Z Li C Xu F. Association between the CD209 promoter−336A/G polymorphism and susceptibility to tuberculosis: a meta-analysis. Respirology. (2012) 17:847–53. 10.1111/j.1440-1843.2012.02185.x 22553928

[197] Ma X Reich RA Wright JA Tooker HR Teeter LD Musser JM. Association between interleukin-8 gene alleles and human susceptibility to tuberculosis disease. J Infect Dis. (2003) 188:349–55. 12870115 10.1086/376559

[198] Selvaraj P Prabhuanand S Jawahar MS Chandra G Banurekha B Narayanan PR. Promoter polymorphism of IL-8 gene and IL-8 production in pulmonary tuberculosis. Curr Sci. (2006) 90:952–4.

[199] Cooke GS Campbell SJ Fielding K Sillah J Manneh K Sirugo G. Interleukin-8 polymorphism is not associated with pulmonary tuberculosis in the gambia. J Infect Dis. (2004) 189:1545–6. 15073694 10.1086/382489

[200] Flores-Villanueva PO Ruiz-Morales JA Song CH Flores LM Jo EK Montaño M. A functional promoter polymorphism in monocyte chemoattractant protein-1 is associated with increased susceptibility to pulmonary tuberculosis. J Exp Med. (2005) 202:1649–58. 16352737 10.1084/jem.20050126 PMC2212957

[201] Jamieson SE Miller EN Black GF Peacock CS Cordell HJ Howson JM. (2012). Evidence for a cluster of genes on chromosome 17q11-q21 controlling susceptibility to tuberculosis and leprosy in Brazilians. Genes Immun (2004) 5:46–57. 14735149 10.1038/sj.gene.6364029

[202] Arji N Busson M Iraqi G Bourkadi JE Benjouad A Boukouaci W. The MCP-1 (CCL2)−2518 GG genotype is associated with protection against pulmonary tuberculosis in Moroccan patients. J Infect Dev Ctries (2012) 6:73–78. 22240432 10.3855/jidc.1925

[203] Thye T Nejentsev S Intemann CD Browne EN Chinbuah MA Gyapong J. MCP-1 promoter variant−362C associated with protection from pulmonary tuberculosis in Ghana, West Africa. Hum Mol Genet (2009) 18:381–8. 10.1093/hmg/ddn352 18940815 PMC2638774

[204] Chu SF Tam CM Wong HS Kam KM Lau YL Chiang AK. Association between CCL5 functional polymorphisms and tuberculosis in Hong Kong Chinese. Genes Immun (2007) 8:475–9. 17625600 10.1038/sj.gene.6364412

[205] Ben-Selma W Harizi H Bougmiza I Ben Kahla I Letaief M Boukadida J. Polymorphisms in the RANTES gene increase susceptibility to active tuberculosis in Tunisia. DNA Cell Biol. (2011) 30:789–800. 10.1089/dna.2010.1200 21510799

[206] Selvaraj P Alagarasu K Singh B Afsal K. CCL5 (RANTES) gene polymorphisms in pulmonary tuberculosis patients of south India. Int J Immunogenet. (2011) 38:397–402. 10.1111/j.1744-313X.2011.01021.x 21707929

[207] Mishra G Poojary SS Raj P Tiwari PK. Genetic polymorphisms of CCL2, CCL5, CCR2 and CCR5 genes in Sahariya tribe of North Central India: an association study with pulmonary tuberculosis. Infect Genet Evol. (2012) 12:1120–27. 10.1016/j.meegid.2012.03.018 22554651

[208] Mhmoud N Fahal A van de Sande WJ. Association of IL-10 and CCL5 single nucleotide polymorphisms with tuberculosis in the Sudanese population. Trop Med Int Health (2013) 18:1119–27. 10.1111/tmi.12141 23790189

[209] Tang NL Fan HP Chang KC Ching JK Kong KP Yew WW. Genetic association between a chemokine gene CXCL-10 (IP-10, interferon gamma inducible protein 10) and susceptibility to tuberculosis. Clin Chim Acta. (2009) 406:98–102. 10.1016/j.cca.2009.06.006 19523460 PMC7124215

[210] Selvaraj P Narayanan PR Reetha AM. Association of functional mutant homozygotes of the mannose binding protein gene with susceptibility to pulmonary tuberculosis in India. Tuber Lung Dis. (1999) 79:221–7. 10692990 10.1054/tuld.1999.0204

[211] Feng FM Guo M Liu Q Wang D Gao BX Sun YH. [Study on mannose-binding protein gene polymorphisms and susceptibility to pulmonary tuberculosis]. Zhonghua Liu Xing Bing Xue Za Zhi. (2006) 27:1082–85. 17415991

[212] Alagarasu K Selvaraj P Swaminathan S Raghavan S Narendran G Narayanan PR. Mannose binding lectin gene variants and susceptibility to tuberculosis in HIV-1 infected patients of South India. Tuberculosis (Edinb). (2007) 87:535–43. 10.1016/j.tube.2007.07.007 17855170

[213] Cosar H Ozkinay F Onay H Bayram N Bakiler AR Anil M. Low levels of mannose-binding lectin confers protection against tuberculosis in Turkish children. Eur J Clin Microbiol Infect Dis. (2008) 27:1165–9. 10.1007/s10096-008-0573-8 18612667

[214] Capparelli R Iannaccone M Palumbo D Medaglia C Moscariello E Russo A. Role played by human mannose-binding lectin polymorphisms in pulmonary tuberculosis. J Infect Dis. (2009) 199: 666–72. 10.1086/596658 19199550

[215] Singla N Gupta D Joshi A Batra N Singh J Birbian N. Association of mannose-binding lectin gene polymorphism with tuberculosis susceptibility and sputum conversion time. Int J Immunogenet. (2012) 39:10–14. 10.1111/j.1744-313X.2011.01047.x 22050925

[216] da Cruz HL da Silva RC Segat L de Carvalho MS Brandão LA Guimarães RL. MBL2 gene polymorphisms and susceptibility to tuberculosis in a northeastern Brazilian population. Infect Genet Evol. (2013) 19:323–29. 10.1016/j.meegid.2013.03.002 23524205

[217] Shi J Xie M Wang JM Xu YJ Xiong WN Liu XS. Mannose-binding lectin two gene polymorphisms and tuberculosis susceptibility in Chinese population: a meta-analysis. J Huazhong Univ Sci Technolog Med Sci. (2013) 33:166–71. 10.1007/s11596-013-1091-1 23592124

[218] Chen M Deng J Su C Li J Wang M Abuaku BK. Impact of passive smoking, cooking with solid fuel exposure, and MBL/MASP-2 gene polymorphism upon susceptibility to tuberculosis. Int J Infect Dis. (2014) 29:1–6. 10.1016/j.ijid.2014.08.010 25312983

[219] Chen M Liang Y Li W Wang M Hu L Abuaku BK. Impact of MBL and MASP-2 gene polymorphism and its interaction on susceptibility to tuberculosis. BMC Infect Dis. (2015) 15:151. 10.1186/s12879-015-0879-y. 25887173 PMC4399571

[220] OzbaS-Gerçeker F Tezcan I Berkel AI Ozkara S Ozcan A Ersoy F. The effect of mannose-binding protein gene polymorphisms in recurrent respiratory system infections in children and lung tuberculosis. Turk J Pediatr. (2003) 45:95–8. 12921293

[221] Thye T Niemann S Walter K Homolka S Intemann CD Chinbuah MA. Variant G57E of mannose binding lectin associated with protection against tuberculosis caused by Mycobacterium africanum but not by. M.tuberculosis. PLoS ONE (2011) 6:e20908. 10.1371/journal.pone.0020908 21695215 PMC3112207

[222] Torrelles JB Azad AK Henning LN Carlson TK Schlesinger LS. Role of C-type lectins in mycobacterial infections. Curr Drug Targets. (2008) 9:102–12. 18288961 10.2174/138945008783502467

[223] Zhang X Jiang F Wei L Li F Liu J Wang C. Polymorphic allele of human MRC1 confer protection against tuberculosis in a Chinese population. Int J Biol Sci. (2012) 8:375–82. 10.7150/ijbs.4047 22393309 PMC3291854

[224] Zhang X Li X Zhang W Wei L Jiang T Chen Z. The novel human MRC1 gene polymorphisms are associated with susceptibility to pulmonary tuberculosis in Chinese Uygur and Kazak populations. Mol Biol Rep. (2013) 40:5073–83. 10.1007/s11033-013-2610-7 23653008

[225] Liu C He T Rong Y Du F Ma D Wei Y. Association of Mannose-binding Lectin Polymorphisms with Tuberculosis Susceptibility among Chinese. Sci Rep. (2016) 6:36488. 10.1038/srep36488 27812036 PMC5095599

[226] Amiri A Sabooteh T Shahsavar F Anbari K Pouremadi F. Mannose-Binding Lectin (*MBL*) gene polymorphisms in susceptibility to pulmonary tuberculosis among the Lur population of Lorestan Province of Iran. Genom Data. (2017) 12:146–50. 10.1016/j.gdata.2017.05.005 28540182 PMC5432655

[227] Torrado E Cooper AM. IL-17 and Th17 cells in tuberculosis. Cytokine Growth Factor Rev. (2010) 21:455–62. 10.1016/j.cytogfr.2010.10.004 21075039 PMC3032416

[228] Yu ZG Wang BZ Li J Ding ZL Wang K. Association between interleukin-17 genetic polymorphisms and tuberculosis susceptibility: an updated meta-analysis. Int J Tuberc Lung Dis. (2017) 21:1307–13. 10.5588/ijtld.17.0345 29297452

[229] Chimusa ER Zaitlen N Daya M Möller M van Helden PD Mulder NJ. Genome-wide association study of ancestryspecific TB risk in the South African Coloured population. Hum Mol Genet. (2014) 23:796–809. 10.1093/hmg/ddt462 24057671 PMC3888262

[230] Curtis J Luo Y Zenner HL Cuchet-Lourenço D Wu C Lo K. Susceptibility to tuberculosis is associated with variants in the ASAP1 gene encoding a regulator of dendritic cell migration. Nat Genet. (2015) 47:523–27. 10.1038/ng.3248 25774636 PMC4414475

[231] Hu X Peng W Chen X Zhao Z Zhang J Zhou J. No significant effect of ASAP1 gene variants on the susceptibility to tuberculosis in Chinese population. Medicine (Baltimore) (2016) 95:e3703. 10.1097/MD.0000000000003703 27227929 PMC4902353

[232] Grant AV Sabri A Abid A Abderrahmani Rhorfi I Benkirane M Souhi H. A genome-wide association study of pulmonary tuberculosis in Morocco. Hum Genet. (2016) 135:299–307. 10.1007/s00439-016-1633-2 26767831 PMC5042142

[233] Sobota RS Stein CM Kodaman N Scheinfeldt LB Maro I Wieland-Alter W. A locus at 5q33.3 confers resistance to tuberculosis in highly susceptible individuals. Am J Hum Genet. (2016) 98:514–24. 10.1016/j.ajhg.2016.01.015 26942285 PMC4800052

[234] Coll F Phelan J Hill-Cawthorne GA Nair MB Mallard K Ali S. Genome-wide analysis of multi- and extensively drug-resistant Mycobacterium tuberculosis. Nat Genet. (2018) 50:307–16. 10.1038/s41588-017-0029-0 29358649

[235] Duncan C Jamieson F Mehaffy C. Preliminary evaluation of exome sequencing to identify genetic markers of susceptibility to tuberculosis disease. BMC Res Notes (2015) 8:750. 10.1186/s13104-015-1740-5 26643661 PMC4672511

[236] Wang Y Yan XY Wu JC Ji GY Wu SQ Huang WW. Analysis of silent information regulator 1 (SIRT1) gene polymorphisms in antituberculosis- drug-induced hepatotoxicity in a prospective cohort study. Int J Clin Pharmacol Ther. (2016) 54:775–81. 10.5414/CP202506 26952036

[237] Weiner M Peloquin C Burman W Luo CC Engle M Prihoda TJ. Effects of tuberculosis, race, and human gene SLCO1B1 polymorphisms on rifampin concentrations. Antimicrob Agents Chemother. (2010) 54:4192–200. 10.1128/AAC.00353-10 20660695 PMC2944564

[238] Chigutsa E Visser ME Swart EC Denti P Pushpakom S Egan D. The SLCO1B1 rs4149032 polymorphism is highly prevalent in South Africans and is associated with reduced rifampin concentrations: dosing implications. Antimicrob Agents Chemother. (2011) 55:4122–7. 10.1128/AAC.01833-10 21709081 PMC3165308

[239] Hennig S Naiker S Reddy T Egan D Kellerman T Wiesner L. Effect of SLCO1B1 polymorphisms on rifabutin pharmacokinetics in African HIVinfected patients with tuberculosis. Antimicrob Agents Chemother. (2015) 60:617–20. 10.1128/AAC.01195-15 26482301 PMC4704238

[240] Petros Z Lee MM Takahashi A Zhang Y Yimer G Habtewold A. Genome-wide association and replication study of anti-tuberculosis drugs-induced liver toxicity. BMC Genom (2017) 17:755. 10.1089/omi.2017.0019 27671213 PMC5037629

[241] Perwitasari DA Irham LM Darmawan E Mulyani UA Atthobari J. CYP2E1 polymorphism, acetylator profiles and drug-induced liver injury incidence of Indonesian tuberculosis patients. Indian J Tuberc. (2016) 63:139–43. 10.1016/j.ijtb.2016.08.001 27865233

[242] Matsumoto T Ohno M Azuma J. Future of pharmacogeneticsbased therapy for tuberculosis. Pharmacogenomics. (2014) 15:601–7. 10.2217/pgs.14.38 24798717

[243] Wallis RS Hafner R. Advancing host-directed therapy for tuberculosis. Nat Rev Immunol. (2015) 15:255–63. 10.1038/nri3813 25765201

